# Forward and inverse optimality problems of bone adaptation at the homogenised RVE level

**DOI:** 10.1007/s10237-025-02024-8

**Published:** 2026-01-13

**Authors:** Philippe K. Zysset

**Affiliations:** ARTORG Center for Biomedical Engineering Research, University of Bern, Bern, Switzerland

**Keywords:** Anisotropic elasticity, Bone adaptation, Bone density, Fabric, Inverse problem, Structural optimisation

## Abstract

Bone was shown to adapt to mechanical loading through the concept of a mechanostat that regulates cell activity to maintain a specific strain signal within the tissue. Current computer models simulate bone resorption and formation in the presence of key biological agents, reproduce a realistic architecture of trabecular bone along principal stresses and estimate changes in bone strength related to immobilisation, overloading, metabolic diseases or drug therapies. However, clinical diagnostics of bone diseases *in vivo* rely primarily on X-ray-based densitometry and computer tomography that do not have the resolution to describe bone microarchitecture in full detail and evaluation of personalised bone strength is therefore based on a homogenised description of bone mechanical properties using density and fabric. Continuum-level bone adaptation theories rely primarily on bone density and do not involve local optimisation principles to predict fabric. The inverse problems of predicting applied loads from bone morphology typically exploit density but not fabric. Accordingly, this work formulates and provides analytical solutions for optimal bone adaptation at the homogeneous, anisotropic RVE level using bone density- and fabric-mechanical property relationships for three different mechanostat criteria. Two of these criteria elicit different adaptive responses for tensile and compressive strains. Forward solutions for density and fabric are provided at a continuum point for a given local stress, while inverse solutions for local stress are derived for given density and fabric for all three criteria. The 3D solutions are specialised to 2D and 1D for comprehension and compared among the different criteria. In the future work, the obtained solutions will enable simple forward simulation of personalised bone adaptation and inverse estimation of bone loading for clinical diagnostic tools such as high-resolution peripheral quantitative computed tomography (HR-pQCT) or photon counting computed tomography (PCCT).

**Supplementary Information:**

The online version contains supplementary material available at 10.1007/s10237-025-02024-8.

## Introduction

### State of the art

Bone is a remarkable hierarchical composite biomaterial primarily made of mineral, type I collagen and water. It is found in compact and trabecular forms and adapts to its mechanical environment. The structural optimisation of the bone diaphyses and the complementary arrangement of compact and trabecular bone with respect to mass in the human skeleton is described in depth by Currey (2002).

Trabecular bone is lighter and more compliant than compact bone and covers a broad range of mechanical properties with a volume fraction (BV/TV) or structural density ($\rho$) extending from 5 to 45%. This makes trabecular bone an attractive solution for load transfer in epiphyses, sandwich design in flat bones and core stability of small bones.

The 1D mechanical properties of bone such as Young’s modulus or yield/ultimate stress behave as power functions of $\rho$ (Carter and Hayes 1977; Rice et al. 1988), but also depend on trabecular or collagen fibre orientation for trabecular or compact bone, respectively (Ashman et al. 1989; Martin and Ishida 1989). Trabecular orientation can be characterised in 2D histological sections using the concept of mean intercept length (MIL) (Whitehouse 1974). The orientation distribution of MIL follows an ellipse with the major axis along the main direction of the trabeculae. The orientation distribution of MIL can be generalised to an ellipsoid in 3D and is described mathematically by a positive definite, second-order fabric tensor (Harrigan and Mann 1984).

Since the early 90’s, 3D microCT reconstructions (Kuhn et al. 1990) allow a detailed 3D characterisation of trabecular architecture (Goulet et al. 1994). Trabecular anisotropy is computed by MIL or other methods such as mean surface length (MSL) from the interfaces of segmented images (Hosseini et al. 2017) or alternatively by the gradient structure tensor (GST) from grey level images (Tabor 2011).

The generalisation of MIL allows the formulation of relationships between structural density, the fabric tensor and the elasticity tensor (Cowin 1985) that were first verified with orthogonal ultrasound measurements (Turner et al. 1990) and then by uniaxial mechanical tests (Snyder and Hayes 1990; Goulet et al. 1994; Zysset and Curnier 1996). Along the same idea, Cowin proposed a relationship between density, the fabric tensor and a Tsai–Wu strength criterion (Cowin 1986), that was later extended to a more general quadric strength criterion (Schwiedrzik et al. 2013).

The ability to solve large linear equation systems on high performance computers opened the microFE era (VanRietbergen 2001). The generally anisotropic apparent stiffness tensor of trabecular bone volume elements of 4-6 mm side length could be computed on parallel CPUs (VanRietbergen et al. 1995). It was found that the general apparent stiffness tensor of trabecular bone can be approximated by orthotropic symmetry (Zysset et al. 1998), which does not contradict the observation of non-orthogonal arrangement of trabeculae (Skedros and Baucom 2007), as the material symmetry is understood in an average, statistical sense.

It was also confirmed that the principal directions of the fabric tensor coincide with the orthotropic axes of symmetry of the apparent stiffness tensor (Odgaard et al. 1997) and fabric–elasticity relationships (Cowin 1985; Zysset and Curnier 1995) could be validated with larger experimental and computational datasets (VanRietbergen et al. 1995; Zysset et al. 1998; VanRietbergen et al. 1998; Kabel et al. 1999; Zysset 2003; Rincon-Kohli and Zysset 2009). Interestingly, the constants of the fabric–elasticity model are close to identical for different anatomical locations such as the proximal femur, distal radius and vertebral body (Gross et al. 2013) suggesting a universal relationship between the architecture achieved by the remodelling process and the functional elastic properties. Moreover, fluctuations of tissue properties related to the different levels of mineralisation within trabecular bone do not affect the apparent elastic properties beyond a few per cent (Gross et al. 2012), emphasising the dominant role of BV/TV and architecture.

However, the computed apparent stiffness tensors of trabecular bone depend rather heavily on boundary conditions (Pahr and Zysset 2008), especially for low volume fraction due to the vanishing representativity of the volume element. A heterogeneity index was introduced to exclude samples that poorly satisfy the assumptions of the underlying homogenisation procedure (Panyasantisuk 2015; Simon et al. 2022).

A broad statistical analysis demonstrated that BV/TV and fabric explain up to 97% of apparent trabecular bone elastic properties computed by microFE (Maquer et al. 2015). A similar result was obtained for apparent yield stresses of trabecular bone (Musy et al. 2017). The relative contribution of fabric was higher for yield stress (23%) than for elastic constants (9%). Most importantly, further architectural indices did not significantly improve these relationships.

Based on the early observation that trabecular bone density increases with stress intensity, Cowin formulated a theory of adaptive elasticity, applied it to small strain and evaluated uniqueness and stability of the model (Cowin and Hegedus 1976; Hegedus and Cowin 1976; Cowin and Nachlinger 1978). A decade later, Carter and others (Carter et al. 1987; Weinans et al. 1992) applied a functional adaptation theory of trabecular bone to a continuum 2D FE model of the proximal femur. Their algorithm consists of a first-order differential equation in time that adapts the bone density distribution in the FE model of a bone subjected to multiple loading cases to reach homeostasis of a biomechanical stimulus selected as a convolution of an effective stress history. Under a few constraints, the simulation reproduces a convincing map of the density of the proximal femur.

Back in the 19th century, Wolff’s law (Wolff 1892) stated that trabecular trajectories follow principal stresses (Meyer 1867). Following the pioneering works of Cowin and Carter, multiple formulations of anisotropic bone remodelling were proposed, e.g. Pettermann et al. (1997). The framework of continuum damage mechanics was exploited to derive an evolution law for the whole anisotropic compliance tensor or for a pseudo-damage tensor expressing trabecular morphology (Jacobs et al. 1997; Doblare and Garcia 2002). Criteria for the biomechanical stimulus were expressed in the associated variables, and the results suggest that the remodelling algorithm indeed aligns the main trabecular orientation with the main principal stresses.

At the micro-architectural level, computation of trabecular bone adaptation was pioneered by Rik Huiskes and his team (Huiskes et al. 2000; Ruimerman et al. 2003). This breakthrough bridged a strain sensing process orchestrated by osteocytes with the anisotropic spatial arrangement of trabeculae that is observed at the whole-bone level. At the tissue level, the selected biomechanical stimulus was strain energy density with a physical unit in MPa. The validity of this concept was confirmed in several animal models such as the compressive loading of the mouse tail (Marques et al. 2023).

Fyhrie and Carter (1986) proposed an optimising principle for trabecular orientation at the continuum level by minimising bone mass for a given homeostasis function such as strain energy density or a failure stress criterion and which solution indeed aligned the orthotropic material axes of trabecular bone with the ones of principal stresses. However, the relationship between the stiffness tensor and the extent of trabecular anisotropy was not exploited. The mathematical formulation of Wolff’s law was also explored by Cowin (1986) where the alignment between fabric, stress and strain results from the property of commutativity of the respective tensors. Applying minimisation of the free energy density for a fixed mass at the local, continuum level, Luo and An (1998) confirmed that the principal fabric tensor directions coincide with the principal stress tensor directions. However, they did not resolve the relationships between fabric eigenvalues and the principal stress components.

Using the Zysset–Curnier fabric–elasticity model (Zysset 2003), Marangalou et al. (2015) postulated an empirical power relationship between fabric eigenvalues and principal stress components in order to estimate fabric from an apparent stress field. They obtained reasonable estimates of fabric in the proximal femur using an iterative finite element simulation with prescribed loads. No optimisation background was presented, and the potential role of the sign of the principal stress components was not discussed.

Following the development of the first remodelling theories (Carter et al. 1987), the idea of an inverse problem emerged to predict *in vivo* loading from bone geometry and density distribution. Fischer et al. applied an optimisation procedure to determine the load intensities on a 2D model of a bone epiphysis (Fischer et al. 1995) and of the human proximal femur (Fischer et al. 1998). They reported that the outcome is not unique, namely that multiple load cases can lead to a similar bone density distribution (Fischer et al. 1996). In these studies, a global optimisation problem is resolved that minimises the sum of the differences of the actual and reference biomechanical stimuli over all the elements of a region of interest in the FE model. More recently, Campoli et al. used artificial neural networks on forward bone remodelling simulation results to estimate the load on a 3D model of the human proximal femur from a CT scan (Campoli et al. 2012). Similarly, Garijo et al. compared linear regression, artificial neural networks and support vector machines for prediction of proximal femur loads from bone density distribution (Garijo et al. 2014). As a first attempt to distinguish tension and compression in the biomechanical stimulus, Schenk and Zysset defined an isotropic compressive strain tensor as homeostasis to predict the load and moments applied on the human distal radius through the solution of a minimisation problem (Schenk and Zysset 2022). However, in all these studies, the material properties of trabecular bone were considered as isotropic and the fabric was not accounted for. In addition, the latter two methods using artificial neural networks require a large database of forward solutions to address the inverse adaptation problem.

This inverse problem was also tackled at the micro-structural level, where Christen et al. applied minimisation of strain energy density variance in microFE analyses to determine the load on a murine model of bone remodelling (Christen et al. 2012). They applied the same methodology to high-resolution peripheral quantitative computed tomography (HR-pQCT) reconstructions to estimate subject-specific load of the human distal radius (Christen et al. 2013). They also established the resolution-dependency, reproducibility and sensitivity of the methodology *in vivo* (Christen et al. 2016). In parallel, Zadpoor et al. used artificial neural networks to predict load from the morphology of a volume element of trabecular bone (Zadpoor et al. 2013). Synek and Pahr examined parameter sensitivity and confirmed plausibility of the strain energy density minimisation for joint load prediction on the human femoral head (Synek and Pahr 2018). Further extension of this methodology consisted in quantifying load changes from the evolution of the bone density distribution along a murine loading experiment (Walle et al. 2021).

The increasing use of the computationally more efficient homogenised FE analysis to compute the strength of the peripheral skeleton in a clinical environment motivates not only the implementation of efficient forward remodelling algorithms but also the resolution of inverse problems looking for the most likely load configuration of a bone from its morphology and internal distribution of volume fraction and fabric (Zadpoor 2013). In an effort to exploit the computationally efficient homogenised FE analysis for the inverse problem, Bachmann et al. (2023, 2023) determined a density power function to homogenise the strain energy density stimulus proposed by Christen et al. (2012) using microFE models of trabecular bone cubes. They obtained reasonable distal radius and hip joint load predictions using homogenised FE, but fabric of trabecular bone and the triaxial state of stress are not exploited in the optimisation scheme.

The above state of the art suggests that several problems in bone adaptation remain open. Specifically, the forward and inverse bone adaptation schemes were not presented as optimality problems at the local, anisotropic continuum level when both density and fabric are taken into account. Moreover, only strain energy density was considered as a stimulus at the tissue level, which is indifferent upon compressive or tensile stresses.

### Aims

Following this introduction, the present study aims in a first part at formulating and resolving a forward bone adaptation problem in terms of minimisation of a convex strain metric at the continuum level of a homogenised representative volume element (RVE) by using analytical density–fabric-mechanical property relationships. A unique bone architecture is derived for an applied stress tensor. Three different convex strain metrics are considered: strain energy density, a yield/damage criterion and principal strain criterion. In a second part, the study aims at formulating and resolving the inverse bone adaptation problem in terms of minimisation of the same metrics to recover the maximal functional stress tensor for a given bone architecture in the RVE.

### Organisation

In the second section, the notion of a representative volume element for trabecular bone is recalled and the fabric–elasticity as well as the fabric–yield relationships used in this work are summarised. The motivation for three different strain metrics, a normalised complementary free energy density, a generalised yield criterion and a principal strain criterion is developed. A mathematical formulation of the forward and inverse problem is proposed in a homogenised, orthotropic bone framework.

In the third section, the forward problem is resolved analytically for three strain metrics for a given functional stress. The solutions for the fabric tensor are computed in 3D, and specialised to the 2D case. A reduction to the 1D case is exploited to examine more specifically the role of density. A global comparison of the solutions of the three metrics is then presented.

In the fourth section, the inverse problem is resolved analytically for the same three metrics. The optimal stresses are again computed in 3D and specialised to the 2D and 1D cases. A global comparison is also included.

In section 5, a discussion summarises the obtained results, exposes the limitations, suggests the potential impact of this work, and ends with a brief conclusion.

### Notations

Hereafter, scalars are denoted by italic letters (e.g. time *t* or density $\rho$), vectors by bold face minuscules (e.g. position $\textbf{x}$), second-order tensors by bold face majuscules (e.g. fabric tensor $\textbf{M}$) and fourth-order tensors by outline majuscules (e.g. $\mathbb {I}$). With these notations, let $\textbf{E}$ denote the Green–Lagrange strain $\textbf{E}=\frac{1}{2}(\textbf{F}^T\textbf{F}-\textbf{I})$ defined from the gradient $\textbf{F}=\nabla _{\textbf{x}}\textbf{y}$ of the motion $\textbf{y}:(\textbf{x},t)\mapsto \textbf{y}(\textbf{x},t)$ and $\textbf{S}$ the conjugate second Piola–Kirchhoff stress defined through the material version of Cauchy’s theorem $\textbf{s}=\textbf{S}\textbf{n}$, where $\textbf{s}=\textbf{F}^{-1}\textbf{p}$ is transformed by the inverse gradient from the nominal stress vector $\textbf{p}$ and $\textbf{n}$ is the original normal vector. $\textbf{S}$ and $\textbf{E}$ are conjugate or dual because their internal power is equal to the external power supplied to the solid: $\int _{\Omega }\,\textbf{S}\pmb {:}{\dot{\textbf{E}}}\;dV\,$= $\int _{\partial \Omega }\,\textbf{p}\pmb {\cdot }{\dot{\textbf{y}}}\;dA$ where $\Omega$ is the solid original form and ${\dot{\textbf{y}}}$ the particle velocity. Both $\textbf{E}$ and $\textbf{S}$ are symmetric and objective, i.e. insensitive to a change of reference frame, which simplifies the direct formulation of constitutive laws in their terms. These strain and stress measures degenerate into their small strain counterparts $\boldsymbol{\epsilon }$ and $\boldsymbol{\sigma }$ in small strains and in the absence of rotations. The reader is referred to Curnier (2004) for complete treatments. Finally, the following tensor products will be used:

$$\begin{aligned} \begin{array}{clll} \pmb {\cdot } & \text {vector dyadic} & (\textbf{a}\otimes \textbf{b})\,\textbf{x}= (\textbf{x}\pmb {\cdot }\textbf{b})\,\textbf{a} ,\quad \forall \textbf{x} & (^i\textbf{e}\!\otimes \!^i\textbf{e})\,\textbf{x}= \textrm{x}_{i}\,^i\textbf{e} \\ \pmb {\cdot } & \text {tensor dyadic} & (\textbf{A}\!\otimes \!\textbf{B})\,\textbf{X}= (\mathbf {X\!\pmb {:}\!B})\,\textbf{A} \!,\, \forall \textbf{X} & (\textbf{I}\!\otimes \!\textbf{I})\,\textbf{X}= (\textrm{tr}\textbf{X})\textbf{I} \\ \pmb {\cdot } & \text {tensor product} & (\textbf{A}\underline{\otimes }\textbf{B})\,\textbf{X}= \textbf{A}\,\textbf{X}\,\textbf{B}^{T} ,\,\, \forall \textbf{X} & (\textbf{I}\underline{\otimes }\textbf{I})\,\textbf{X}= \textbf{X} \\ \pmb {\cdot } & \text {transp. product} & [\textbf{A} \overline{\otimes } \textbf{B}]\,\textbf{X}= \textbf{A}\,\textbf{X}^{T}\,\textbf{B}^{T},\, \forall \textbf{X} & (\textbf{I} \overline{\otimes } \textbf{I})\,\textbf{X}= \textbf{X}^{T} \\ \pmb {\cdot } & \text {symm. product} & (\textbf{A}\underline{\overline{\otimes }}\textbf{B})\,\textbf{X}= \textbf{A}\,\textbf{X}\,\textbf{B}^{T} \!, \forall \textbf{X}\!=\!\textbf{X}^{T} & (\textbf{I}\underline{\overline{\otimes }}\textbf{I})\,\textbf{X}= \textbf{X} \end{array} \end{aligned}$$

hence $$\textbf{A} \,\underline{\overline{\otimes }}\, \textbf{B} = \begin{array}{c} \!\!\frac{{1}}{{2}}\!\! \end{array}\, \big ( \textbf{A} \,\underline{\otimes }\, \textbf{B} + \textbf{A}\, \overline{\otimes }\, \textbf{B} \big )$$. The summation convention on repeated indexes is used. The spectral decomposition of the material strain and stress is expressed by $\textbf{E}=\varepsilon _i ({^i\textbf{e}}\otimes {^i\textbf{e}})$ and $\textbf{S}=\sigma _i ({^i\textbf{s}}\otimes {^i\textbf{s}})$ where $\sigma _i$ and $\varepsilon _i$ are the stress and strain eigenvalues.

## Density and fabric-mechanical property relationships

### Density and fabric

The continuum assumption for trabecular bone morphology was estimated to 2-3 times the trabecular spacing (Harrigan et al. 1988), but this appeared insufficient for elastic properties where a RVE of approximately 5x5x5 mm represented a better compromise (Zysset et al. 1998). Larger volume elements often present morphological heterogeneity and are limited by human anatomy.

The primary determinant of trabecular bone mechanical properties is structural density $\rho$ also named bone volume over total volume (BV/TV). This positive variable belongs to the interval [0, 1], 0 reflecting the absence of bone and 1 fully compact bone tissue. In fact, trabecular bone exhibits a volume fraction between 5 and 45% (Harrigan et al. 1988; Zysset et al. 1998; Daszkiewicz et al. 2017). Similarly to cellular solids, the functional dependence of elastic modulus, yield stress and ultimate stress of trabecular bone is a monotonic function of $\rho$, f($\rho$) that is typically a power function with an exponent *k* between 1 and 3 (Gibson 1985; Rice et al. 1988; Zysset et al. 1998, 1999; Wili et al. 2017; Fleps et al. 2020).

The second determinant of trabecular bone mechanical properties appearing when considering 3D properties along various orientations is the fabric tensor $\textbf{M}$:

1 $$\begin{aligned} \textbf{M}={m_i}{^i\textbf{M}}\otimes {^i\textbf{M}} \qquad {^i\textbf{M}}={^i\textbf{m}}\otimes {^i\textbf{m}} \quad i=1,d \end{aligned}$$

where ${m_i}$ are the positive fabric eigenvalues and ${^i\textbf{m}}$ the orthonormal, unit fabric eigenvectors with ${^i\textbf{m}}\cdot {^j\textbf{m}}=\delta _{ij}$ and *d* is the dimension of the considered geometrical space (2 or 3). The fabric tensor is usually normed with $\textrm{tr} \textbf{M}=d$ in order to make it independent from $\rho$. This normalisation is supported by the absence of a substantial correlation between density and degree of anisotropy at least in trabecular bone. An alternative normalisation is $\det \textbf{M}=1$, but most of the available results are for $\textrm{tr} \textbf{M}=d$.

Without loss of generality, the fabric eigenvalues may be labelled along their increasing values :

2 $$\begin{aligned} 0 \le {m_1} \le {m_2} \le {m_3} \qquad \sum _{i}^d m_i = d \end{aligned}$$

The degree of anisotropy is defined by the ratio of the largest versus the lowest eigenvalue $DA= {m_d}/{m_1}$ and is not affected by the scaling of the trace. Normalisation makes the resulting tensor dimensionless, but does not account for different sensitivities to structural anisotropy by different morphometric methods. In the frame of a second-order approximation, the eigenvectors of different $\textbf{M}$ must coincide and the ranking of the eigenvalues as well. A practical and efficient way to relate fabric tensors obtained from different methods is the use of a power function (Larsson et al. 2014):

3 $$\begin{aligned} \textbf{M}=\frac{d}{\textrm{tr}\widetilde{\textbf{M}}^n}\widetilde{\textbf{M}}^n \qquad {m_i}=\frac{d}{\textrm{tr}\widetilde{\textbf{M}}^n} {\tilde{m}_i}^n \quad \quad i=1,d \end{aligned}$$

The eigenvalues $m_i$ and the degree of anisotropy DA follow the same power function. The normed fabric tensor $\textbf{M}$ can therefore be used as a second-order approximation of structural anisotropy without consideration of the underlying method employed to quantify it. Nevertheless, a calibration is necessary to fit the single parameter *n* of the transformation.

### Fabric−elasticity relationships

The simple, original fabric-based 4th-order stiffness tensor by Zysset and Curnier (1995) proposed for trabecular bone is expressed by

4 $$\begin{aligned} \mathbb {S}(\rho ,{\textbf {M}})= f(\rho ) \Big (\lambda _0 \, \textbf{M} \otimes \textbf{M} +2\mu _0 \, \textbf{M} \underline{\overline{\otimes }} \textbf{M} \Big ) \end{aligned}$$

where $\rho$ is bone volume fraction, $\lambda _0>0$ and $\mu _0>0$ are strictly positive Lamé constants for the isotropic material when $\rho =1$ and $\textbf{M}=\textbf{I}$. When two fabric eigenvalues degenerate, the model exhibits transverse isotropic symmetry, while when all three eigenvalues are distinct the model exhibits orthotropy with planes of symmetry normal to the three fabric eigenvectors. A key property but also limitation of the model is the multiplicative effect of density and fabric that implies that density scales but does not interfere with the anisotropic tensorial nature of stiffness.

The corresponding simple compliance tensor is expressed by

5 $$\begin{aligned} \mathbb {E}(\rho ,{\textbf {M}})= \frac{1}{f(\rho )} \Big (-\frac{\nu _0}{\epsilon _0} \, \textbf{M}^{-1} \otimes \textbf{M}^{-1} +\frac{1+\nu _0}{\epsilon _0} \, \textbf{M}^{-1}\underline{\overline{\otimes }} \textbf{M}^{-1} \Big ) \end{aligned}$$

where the Lamé constants are related to corresponding Young’s modulus and Poisson’s ratio:

6 $$\begin{aligned} \lambda _0=\frac{\epsilon _0 \nu _0 }{(1+\nu _0)(1-(d-1)\nu _0)} \qquad \mu _0=\frac{\epsilon _0}{2(1+\nu _0)} \end{aligned}$$

and $\textbf{M} \textbf{M}^{-1}=\textbf{I}$. The complementary free energy density (CFE) $\psi ^*$ is defined by

7 $$\begin{aligned} \psi ^*(\textbf{S};\rho ,\textbf{M})=\frac{1}{2} \textbf{S}: \mathbb {E}(\rho ,{\textbf {M}})\textbf{S} \end{aligned}$$

**Fig. 1:**
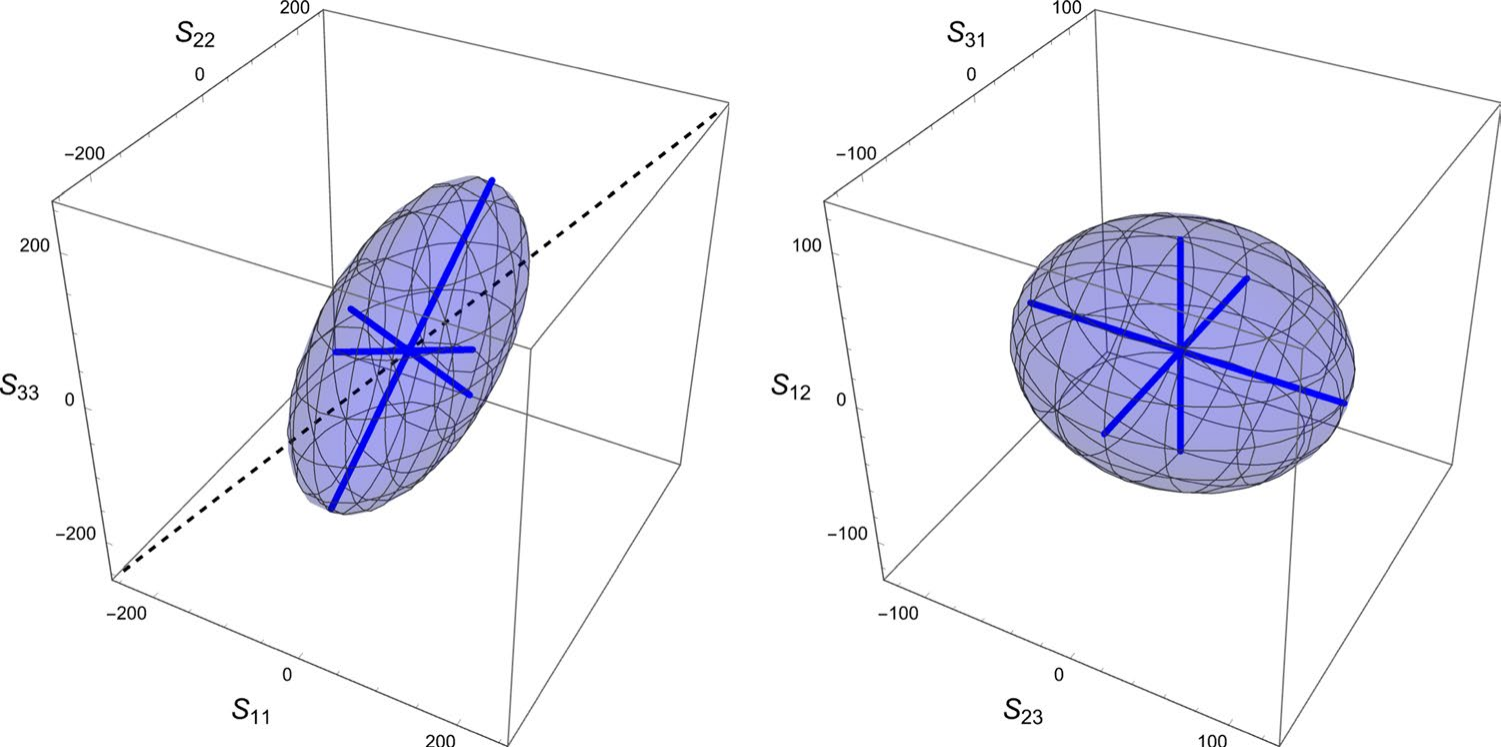
The two components of the complementary free energy density (CFE) in their respective normal and shear stress space. Due to orthotropic symmetry, the two components are uncoupled, and the quadratic nature of the CFE implies that both surfaces are ellipsoidal. However, unlike for the shear components, the principal directions of the ellipsoid are not aligned with the planes of orthotropic symmetry in the normal stress space. The length of the axes correspond to the square root of the inverse of the eigenvalues of the compliance tensor

Due to orthotropic symmetry, the shear stresses are uncoupled from the normal stresses and the CFE is constituted of the sum of two independent terms displayed in Fig. 1. In this compact formulation, the dependence of the anisotropic elastic properties with respect to the fabric eigenvalues is quadratic. A set of simple material constants for trabecular bone that satisfy the isotropy Eq. 6 with the choice $f(\rho )=\rho ^k$ is provided in Table 1 that will be used for illustration of this paper’s results.

**Table 1:** Representative elastic constants for a simple, approximate model of trabecular bone that will be used for illustration of the 3D results of this work

Variables	$\epsilon _0$	$\nu _0$	$\mu _0$	*k*
Units	[MPa]	[-]	[MPa]	[-]
Values	10’000	0.25	4’000	2

### Fabric−yield and fabric-strength relationships

A quadric yield criterion that is non-symmetric in tension and compression was proposed in Schwiedrzik et al. (2013)

8 $$\begin{aligned} y(\textbf{S}; \rho , \textbf{M})=\sqrt{\textbf{S}:\mathbb {F}\textbf{S}}+\textbf{F}:\textbf{S}-1=0 \end{aligned}$$

where $\textbf{F}$ and $\mathbb {F}$ are second- and fourth-order tensors depending on $\rho ,\textbf{M}$ and material constants characterising the yield surface.

The compact form of the fabric-based tensors can be expressed by

9 $$\begin{aligned} \textbf{F}(\rho ,\textbf{M}) =\frac{1}{\hat{f}(\rho )}\frac{1}{2}(\frac{1}{\sigma _0^+}-\frac{1}{\sigma _0^-}) \textbf{M}^{-2}=\frac{f_0}{\hat{f}(\rho )} \textbf{M}^{-2} \end{aligned}$$

10 $$\begin{aligned} \mathbb {F}(\rho , \textbf{M})= & \frac{1}{\hat{f}^2(\rho )} \frac{1}{4}(\frac{1}{\sigma _0^+}+\frac{1}{\sigma _0^-})^2 \Big ( -\zeta _0\;\textbf{M}^{-2} \otimes \textbf{M}^{-2} \nonumber \\+ & (\zeta _0+1)\textbf{M}^{-2} \, \underline{\overline{\otimes }} \, \textbf{M}^{-2} \Big ) \nonumber \\= & \frac{F_0^2}{\hat{f}^2(\rho )} \left( -\zeta _0\;\textbf{M}^{-2} \otimes \textbf{M}^{-2} +(\zeta _0+1)\textbf{M}^{-2} \, \underline{\overline{\otimes }} \, \textbf{M}^{-2}\right) \end{aligned}$$

where $\sigma _0^-$, $\sigma _0^+$ are the isotropic uniaxial yield or ultimate stresses in tension and compression, respectively, $\zeta _0$ characterises the shape of the surface, and

11 $$\begin{aligned} f_0=\frac{1}{2}(\frac{1}{\sigma _0^+}-\frac{1}{\sigma _0^-}) \qquad F_0= \frac{1}{2}(\frac{1}{\sigma _0^+}+\frac{1}{\sigma _0^-}) \end{aligned}$$

The density function $\hat{f}(\rho )$ may be slightly different from $f(\rho )$, the one used for the elastic properties, but remains uncoupled with respect to fabric. In general, the ratio $h(\rho )=\hat{f}(\rho )/f(\rho )$ is a weakly decreasing function of $\rho$. The compact model degenerates into an isotropic model when the fabric tensor becomes identity and the yield shear stress is related to the other material constants by

12 $$\begin{aligned} \tau _0=\frac{1}{F_0}\sqrt{\frac{1}{2(1+\zeta _0)}} \end{aligned}$$

**Fig. 2:**
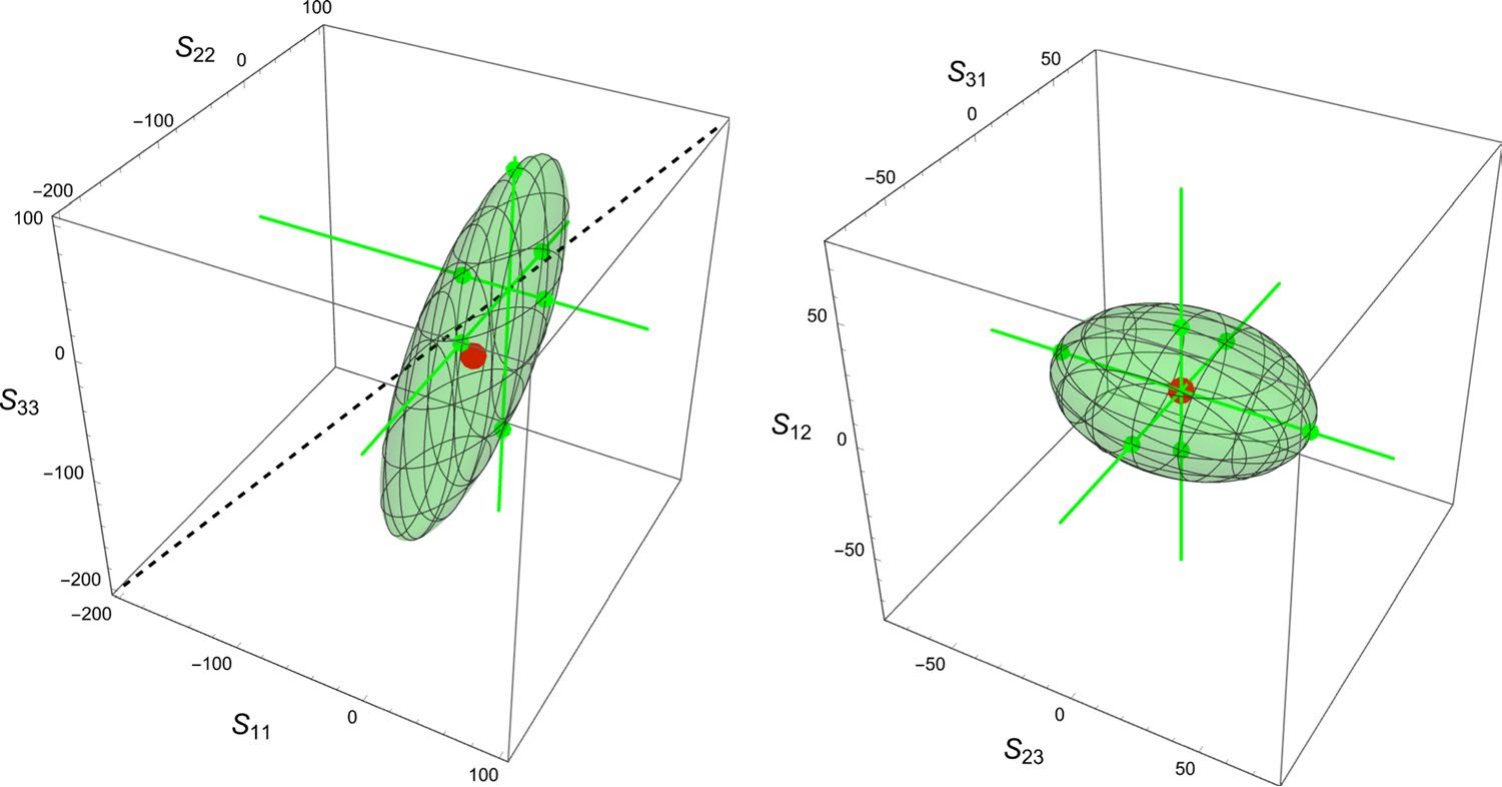
Visualisation of the yield surface for the compact material constants in the uncoupled normal stress space and shear stress space with $\rho =1.0, m_1=0.7, m_2=1.0, m_3=1.3$. The red point is the origin, the dashed line is the trisectrix corresponding to hydrostatic loading. The green points represent the uniaxial yield and shear stresses along the three material directions. Units are MPa

A set of simple material constants for trabecular bone that satisfy the isotropy Eq. 12 with the choice $\hat{f}(\rho )=\rho ^p$ is provided in Table 2 that will be used for illustration of this paper’s results (Fig. 2).

**Table 2:** Compact quadric yield constants that will be used for illustration

Variables	$\sigma _0^+$	$\sigma _0^-$	$\zeta _0$	$\tau _0$	*p*
Units	[MPa]	[MPa]	[-]	[MPa]	[-]
Values	54	72	0.30	38.27	2

### Homogenised bone adaptation

Bone adaptation is a fascinating topic that attracted scientist’s interest since the 19th century and consists of the general concept that bone shape, porosity, structural organisation and to some extent composition is closely adapted to its mechanical function (Wolff 1892; Roux 1895; Currey 2002). The original observation of the trabecular architecture in the proximal femur following the principal stresses in a loaded crane initiated a long history of speculations and research on this topic (Pauwels 1980).

**Fig. 3:**
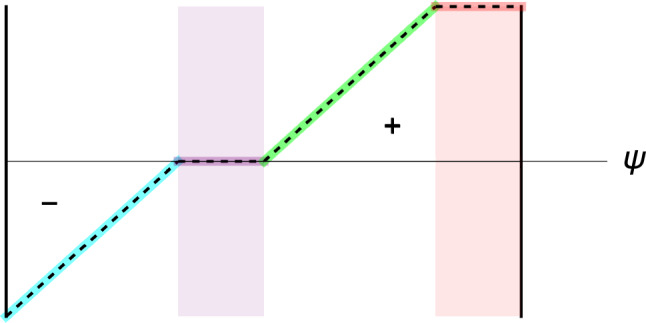
Simplified remodelling rule following (Frost 2003) with a resorption branch (cyan), a lazy zone (violet), an anabolic zone (green) and an overloading or damage zone (red). The y-axis corresponds to the negative, zero or positive rate of change in bone mass, while the x-axis stands for the level of the positive mechanical stimulus $\Psi$, typically the absolute value of the strain amplitude in 1D

Looking for a mechanistic explanation, Harold Frost proposed the "mechanostat" (Fig. 3), where bone formation occurs in a specific strain range, while bone resorption occurs when the strain is too low (Frost 1987, 2003). A lazy zone without net gain of bone mass was also suggested for a range of homeostatic strains, but the existence of this zone was disputed by computational results in humans (Christen et al. 2014). An overloading zone corresponds to an excessive strain that will drive the stimulus back to zero beyond at a maximum failure strain value and lead to resorption of the biomechanically uncoupled bone tissue.

Following these ideas, Huiskes et al. (2000) established a bone remodelling model at the level of the extracellular matrix (ECM) where strain energy density triggers the activity of osteocytes that in turn orchestrate bone resorption and formation. The computational implementation of this theory delivered convincing transformations of bone architectures without clarifying the exact nature of the mechano-transduction agent.

The macroscopic observation that trabecular architecture aligns with applied principal stresses emerges naturally from Huiskes’ bone adaptation model using a strain metric at the bone ECM level. Along this homeostatic rule, non-loaded bone resorbs and bone ECM is used parsimoniously and arranged adequately to experience a suitable mechanical stimulation.

On the one hand, nutrition of the osteocytes was shown to require convected fluid flow in the lacuno-canalicular network produced by volumetric strains of the ECM Weinbaum et al. (1994); Knothe-Tate (2003). On the other hand, excessive tensile or shear strains lead to damage of the ECM and disrupt the lacuno-canalicular network and may trigger a negative signal (Prendergast and Taylor 1994). The probable role of fluid flow in bone remodelling explains why static strains do not seem to have a strong influence and suggests that strain rate may be the key variable that drives the adaptation process (Lanyon and Rubin 1984). Nevertheless, strain amplitude and strain rate correlate highly in daily activities, which explains why strain-based bone remodelling models often prove satisfactory.

In the perspective of clinical applications increasingly based on homogenised FE models, the question arises how to homogenise local mechanical signals into continuous RVEs of trabecular bone in order to obtain meaningful relationships between density, fabric and the applied stresses.

Due to the lack of knowledge about strain type and direction in 3D, the mechanostat underlying turnover was often assigned to strain energy density (SED) as a global metric that incorporates volumetric and deviatoric (shear) strains in all orientations (Carter et al. 1988). However, Lanyon and colleagues (Lanyon et al. 1979) suggested that mechano-transduction in bone may differ in tension and compression, which is a known feature of damage in bone ECM that is not accounted for by SED. For this reason, it may be useful to consider alternative mechanostats that account for this difference in tension and compression and a yield criterion that corresponds to micro-cracking and disruption of the lacuno-canalicular network may be an appropriate candidate. Then, the most natural extension of the 1D strains used to describe Frost’s mechanostat into 2D or 3D bone volume elements are principal strains, and the hypothesis is made here that principal strains with distinct set-points in tension and compression represent a third candidate to characterise 3D mechanical signals responsible for bone turnover and adaptation. Finally, the mechanical stimuli regulating the transition from resorption to formation may be distinct from the one characterising the disruption of the ECM and its lacuno-canalicular network. This represents an additional incentive to explore different mechanostats that drive bone adaptation at the homogenised level.

A forward and an inverse problem of homogenised trabecular bone adaptation are now defined at the RVE level:

- Given $\textbf{S}$ we look for density $\rho$ and fabric $\textbf{M}$ such that a given mechanostat set-point is satisfied with a minimal $\rho$
- Given density $\rho$ and fabric $\textbf{M}$ we look for a stress tensor $\textbf{S}$ such that a given mechanostat set-point is satisfied with a maximal stress intensity

The aim of the next two sections is to formulate and resolve these two optimisation problems using the three different mechanostat (or strain metrics) introduced in the above paragraph.

## Forward problem

In this section, three different mechanical stimuli, the normalised complementary free energy density (CFE), the yield surface *y* and principal strains $\varepsilon _i$ are investigated. The corresponding forward problems will be formulated, the solutions derived analytically and presented graphically. For the sake of completeness and clarity, the solutions will be specialised to 2- and 1-dimensional cases. Finally, the solutions of the three different strain metrics will be compared qualitatively and quantitatively.

### Complementary free energy density

#### Formulation

The forward problem is first addressed with a normalised complementary free energy density (CFE) as strain metric. The normalisation is applied with the monotonically increasing function of density $f(\rho )$ appearing in Eq. 4:

13 $$\begin{aligned} \widehat{\psi ^*}(\textbf{S};\rho ,\textbf{M})=\frac{1}{2 f(\rho )}\textbf{S}:\mathbb {E}(\rho ,\textbf{M})\textbf{S}=\frac{1}{2 f^2(\rho )}\textbf{S}:\mathbb {E}(1,\textbf{M})\textbf{S}=\widehat{\psi ^*_{set}} \end{aligned}$$

The CFE is numerically equivalent to the free energy density $\widehat{\psi }(\textbf{E};\rho ,\textbf{M})$ with the same normalisation. Since $\mathbb {S}(\rho ,\textbf{M})=f(\rho )\mathbb {S}(1,\textbf{M})$ we have

14 $$\begin{aligned} \widehat{\psi ^*}(\textbf{S};\rho ,\textbf{M})= & \hat{\psi }(\textbf{E};\rho ,\textbf{M}) \nonumber \\= & \frac{1}{2 f(\rho )}\textbf{E}:\mathbb {S}(\rho ,\textbf{M})\textbf{E} \nonumber \\= & \frac{1}{2}\textbf{E}:\mathbb {S}(1,\textbf{M})\textbf{E} =\widehat{\psi ^*_{set}} \end{aligned}$$

In fact, the normalised CFE represents a quadratic metric of the strain tensor $\textbf{E}$ at the tissue level. However, as a function of stress, the normalised CFE depends on density at the tissue level:

15 $$\begin{aligned} \widehat{\psi ^*}(\textbf{S};1,\textbf{M})=f^2(\rho )\widehat{\psi ^*}(\textbf{S};\rho ,\textbf{M})=f^2(\rho )\widehat{\psi ^*_{set}} \end{aligned}$$

**Fig. 4:**
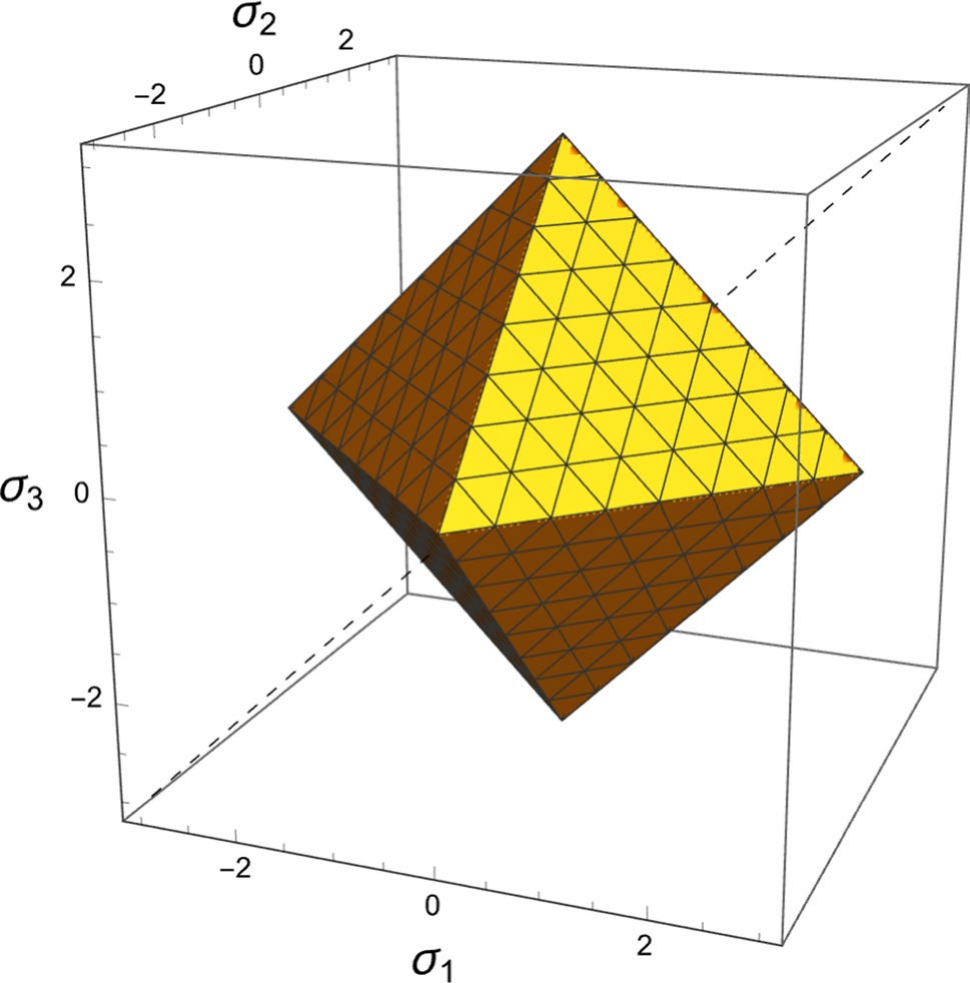
The chosen 3D stress norm $\lambda _S=1$ corresponds to a pyramidal surface in the principal stress space and contains the unit isotropic tension and compression $\textbf{S}=\pm \textbf{I}$ along the dashed trisectrix

Given the homogeneity of degree one of the normalisation of the fabric tensor ($\textrm{tr}(|\lambda |\textbf{M})=|\lambda | \textrm{tr}\textbf{M}$), we choose the following normalisation of the stress tensor $\textbf{S}$:

16 $$\begin{aligned} \hat{\textbf{S}}=\frac{3}{\textrm{tr} |\textbf{S}|}\textbf{S}=\frac{3}{|\sigma _1|+|\sigma _2|+|\sigma _3|}\textbf{S}=\frac{1}{\lambda _S}\textbf{S} \end{aligned}$$

that has the property $\widehat{\lambda \textbf{S}}=\frac{\lambda }{|\lambda |} \hat{\textbf{S}}$ (Fig. 4).

The CFE is homogeneous of degree two with respect to stress

17 $$\begin{aligned} \widehat{\psi ^*}(\lambda \textbf{S};\rho ,\textbf{M})=\lambda ^2\widehat{\psi ^*}(\textbf{S};\rho ,\textbf{M}) \end{aligned}$$

Exploiting this homogeneity property to express the CFE with respect to the normalised stress $\hat{\textbf{S}}$, the set-point Eq. 14 becomes

18 $$\begin{aligned} \widehat{\psi ^*}(\hat{\textbf{S}};1,\textbf{M})=\widehat{\psi ^*}_{set} \left( \frac{f(\rho )}{\lambda _S}\right) ^2=\widehat{\psi ^*_{set}}\lambda _{\rho }^2 \end{aligned}$$

where $\lambda _{\rho }=f(\rho )/ \lambda _S=\sqrt{\widehat{\psi ^*}(\hat{\textbf{S}};1,\textbf{M})/\widehat{\psi ^*_{set}}}$ is a ratio of density with respect to the intensity of the stress tensor. Since the density function $f(\rho )$ and its inverse are monotonic, an optimal fabric tensor $\overline{\textbf{M}}$ may therefore be sought to minimise density $\rho$ for a given stress intensity $\lambda _S$ and orientation $\hat{\textbf{S}}$

19 $$\begin{aligned} \overline{\textbf{M}} = \textrm{Arg Min}_{\{\textrm{tr}\textbf{M}=3\}} \; \widehat{\psi ^*}(\hat{\textbf{S}};1,\textbf{M}) \end{aligned}$$

Knowing the stress amplitude $\lambda _S$ and finding $\overline{\lambda _{\rho }}$ from the above minimisation we can then compute density

20 $$\begin{aligned} \overline{\rho }=f^{-1}\left( \overline{\lambda _{\rho }}\lambda _S\right) \end{aligned}$$

Since $f(\rho )\in [0,1]$, no solution is obtained for $\lambda _S>\overline{\lambda _{\rho }}^{-1}$. For the sake of space, resolution of the problem and discussion of existence/unicity of the solution are provided in subsection 3.1 of supplementary material. References to Eq. or Fig. of supplementary material start with an "S".

#### Solution

**Fig. 5:**
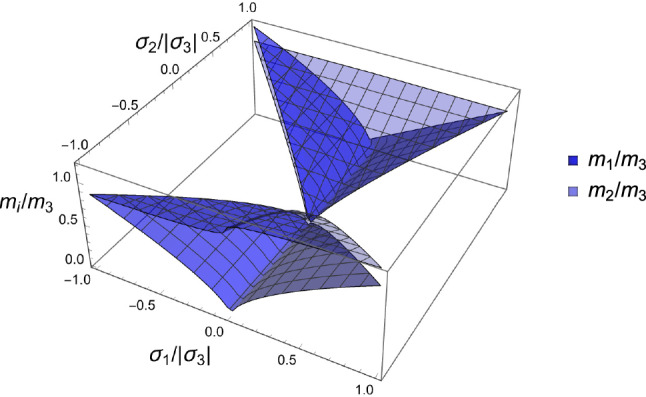
The fabric ratios versus principal stress ratios for the butterfly-shaped domain $|\sigma _1/\sigma _3|\le |\sigma _2/\sigma _3|$ that minimise the complementary free energy density for $\rho =1$. As observed in the skeleton, the larger fabric is usually oriented along the larger stress amplitude, but the solutions differ when the signs of the principal stress ratios are mixed. The ratios $m_1/m_3$ and $m_2/m_3$ are equal on the diagonal $\sigma _1/\sigma _3=\sigma _2/\sigma _3$ and $m_1/m_3=m_2/m_3=1$ when $\sigma _1/\sigma _3=\sigma _2/\sigma _3=1$

The solution for $m_1/m_3$ and $m_2/m_3$ is indeed unique and is depicted in Fig 5.

**Fig. 6:**
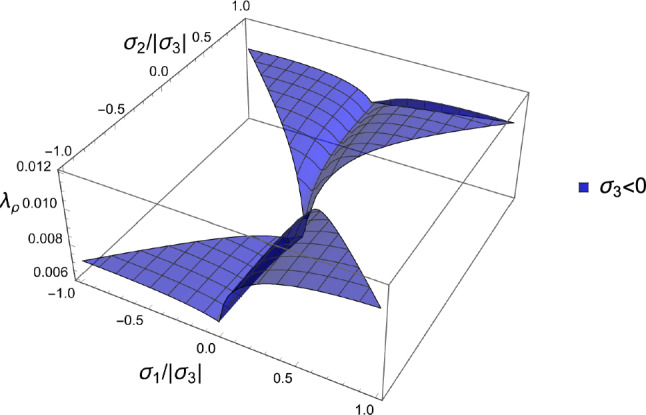
The minimum normalised complementary free energy density (CFE) that results from the optimal fabric ratios versus principal stress ratios. The numerical values of CFE are relative to the $\widehat{\psi ^*_{set}}$. For stress ratios closer to 1 or -1, identical signs of the principal stresses $\{-,-,-\}$ or $\{+,+,+\}$ lead to the lowest CFE

We observe that $\widehat{\psi ^*}(-\hat{\textbf{S}};1,\textbf{M})=\widehat{\psi ^*}(\hat{\textbf{S}};1,\textbf{M})$ which is reflected in Eq. S.12 that is invariant with respect to a simultaneous switch of sign of all principal stresses. The CFE versus $\widehat{\psi }_{set}$ or $\lambda _{\rho }$ computed with the optimal fabric shown in Fig. 6 is lower in the quadrant where all principal stresses have the same sign and is lower towards uniaxial stresses at the centre of the plot. Following Eq. 20, this observation extends to density as it is a monotonic function of the CFE. Due to the chosen normalisation with $f(\rho )$, the solution is independent of density in the conjugate strain space. Interestingly, the solution does not depend on the shear modulus $\mu _0$ as the minimum is achieved in the material coordinate system where the shear components disappear.

#### The 2D case

We observe in Eq. S.12 that $\hat{\sigma }_i=0 \implies m_i=0$, which reduces the dimensionality of the problem. In case $\hat{\sigma }_1=0$, the fabric tensor $\textbf{M}$ becomes of rank 2 and Eq. S.2 reduce to 3 scalar Eq. for 2 fabric eigenvalues and the multiplier $\lambda _{\psi }$:

21 $$\begin{aligned} \frac{\nu }{\epsilon } \left( \sum _{k=2}^3 \frac{\hat{\sigma }_k}{m_k}\right) \hat{\sigma }_i -\frac{(1+\nu )}{\epsilon } \frac{\hat{\sigma }_i^2}{m_i} - \lambda _{\psi }m_i^2= & 0 \qquad i=2,3 \nonumber \\ \sum _{k=2}^3 m_k= & 2 \end{aligned}$$

**Fig. 7:**
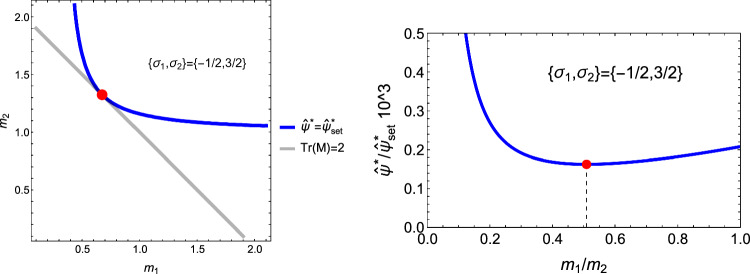
Left: the optimal fabric ratio for a given stress ratio. Right: the CFE with respect to principal stress ratio. The relative value with respect to the one of a uniaxial stress is reported. The same sign of the principal stresses leads to a lower CFE and therefore a lower bone density $\rho$ with respect to stress intensity

**Fig. 8:**
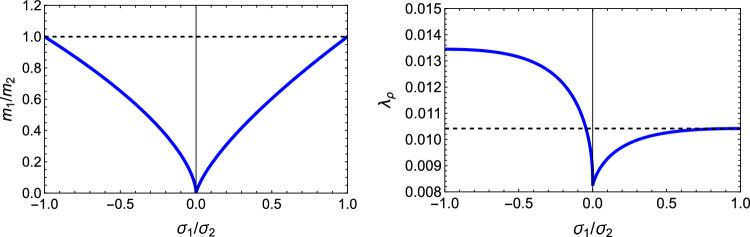
Left: the optimal fabric ratio for a given stress ratio. Right: the CFE with respect to principal stress ratio. The relative value with respect to the one of a uniaxial stress is reported. The same sign of the principal stresses leads to a lower CFE and therefore a lower bone density $\rho$ with respect to stress intensity

The normalisation of the fabric and stress tensors is adapted to their reduced rank so that they degenerate in the 2D identity tensor $\textbf{I}$ when the ratio of the two eigenvalues is 1. A particular solution for the minimum is shown in Fig 7, while the general solution for the stress ratios is plotted in Fig. 8.

#### The 1D case

This is the case $\sigma _1=0$, $\sigma _2=0$ and $\textbf{M}={^3\textbf{e}} \otimes {^3\textbf{e}}$. The CFE becomes

22 $$\begin{aligned} \hat{\psi }^*_{set}=\frac{1}{2}\epsilon _0 E^2=\frac{1}{2\epsilon _0}\left( \frac{\lambda _S}{f(\rho )}\right) ^2=\frac{1}{2\epsilon _0 \lambda _{\rho }^2} \end{aligned}$$

**Fig. 9:**
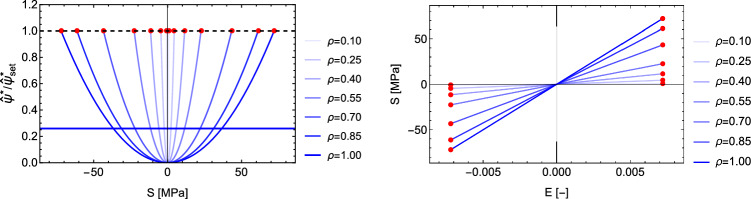
Left: normalised complementary free energy density for densities from 0 to 1. Right: the corresponding stress–strain relationships. The red dots coincide with the set-point

For an elastic modulus of 10 GPa, $\hat{\psi }^*_{set}=0.2592$ leads to a constant homeostatic strain of $\pm 0.0072$ and the stress intensity $\lambda _S$ scales with the density function $f(\rho )$ to remain on the set-point (Fig. 9). As desired, the 1D case degenerates into the original, strain-based mechanostat.

### Generalised yield criterion

#### Formulation

As suggested by Fyhrie and Carter (1986), an alternative model could be the strain metric expressed as a level of damage. We consider here the generalised anisotropic yield criterion (GYC) of Schwiedrzik and Zysset (2013) described above.

23 $$\begin{aligned} y(\textbf{S};\rho , \textbf{M})=\sqrt{\textbf{S}:\mathbb {F}(\rho ,\textbf{M})\textbf{S}}+\textbf{F}(\rho ,\textbf{M}):\textbf{S}=y_{set} \end{aligned}$$

with $y_{set} \in [0,1]$ representing different levels of yield or damage. Here the damage function is homogeneous of degree one with respect to the stress tensor $y(\lambda \textbf{S};\rho ,\textbf{M})=\lambda y(\textbf{S};\rho ,\textbf{M}) \quad \forall \lambda>0$ and the same normalisation of stress (Eq. 16) is applied. Since

24 $$\begin{aligned} \textbf{F}(\rho ,\textbf{M})= & \frac{1}{\hat{f}(\rho )}\textbf{F}(1,\textbf{M}) \nonumber \\ \mathbb {F}(\rho ,\textbf{M})= & \frac{1}{\hat{f}^2(\rho )}\mathbb {F}(1,\textbf{M}) \end{aligned}$$

we observe that $y(\textbf{S};1,\textbf{M})=\hat{f}(\rho )y(\textbf{S};\rho ,\textbf{M})$. Together with the above normalisation of the stress tensor, the damage set-point equation becomes

25 $$\begin{aligned} y(\hat{\textbf{S}};1,\textbf{M})=y_{set}\frac{\hat{f}(\rho )}{\lambda _S}=y_{set} \lambda _{\rho } \end{aligned}$$

where $\lambda _{\rho }=\hat{f}(\rho )/\lambda _S$ is the density versus stress intensity factor. Minimisation of density with respect to the stress intensity implies minimisation of the damage function over $\textbf{M}$:

26 $$\begin{aligned} \overline{\textbf{M}} = \textrm{Arg Min}_{\{\textrm{tr}\textbf{M}=3\}} \; y(\hat{\textbf{S}};1,\textbf{M}) \end{aligned}$$

The minimum delivers

27 $$\begin{aligned} \overline{\lambda _{\rho }} =\frac{y(\hat{\textbf{S}};1,\overline{\textbf{M}})}{y_{set}} \end{aligned}$$

However, $y(-\hat{\textbf{S}};1,\textbf{M}) \ne y(\hat{\textbf{S}};1,\textbf{M})$ for $\hat{\textbf{S}}\ne \textbf{0}$. Accordingly, the minimisation of the function leads to different solutions depending on the sign of the stress eigenvalues. Density is finally obtained

28 $$\begin{aligned} \overline{\rho }=\hat{f}^{-1}\Big ( \lambda _S\frac{y(\hat{\textbf{S}};1,\overline{\textbf{M}})}{y_{set}}\Big )=\hat{f}^{-1}(\overline{\lambda _{\rho }}\lambda _S) \end{aligned}$$

Again, no solution can be found for $\lambda _S>\overline{\lambda _{\rho }}^{-1}$. The resolution and the discussion of existence/unicity of the solution are presented in subsection 3.2 of supplementary material.

#### Solution

**Fig. 10:**
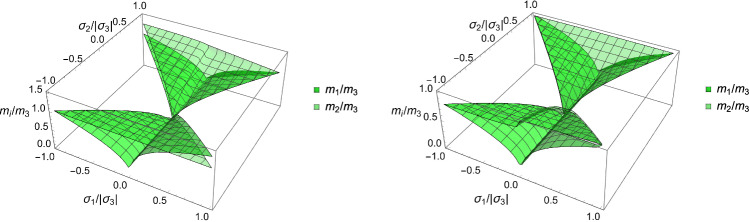
The fabric eigenvalue ratios as a function of the principal stress ratios for a negative (left) and positive (right) principal stress along direction 3

A particular solution of the minimisation problem is shown for a specific stress tensor $\hat{\textbf{S}}$ in the principal strain space in Fig. S2. The solutions for the fabric ratios are shown in Fig. 10. Unlike CFE that is symmetric for opposed stresses, distinction must be made here between negative and positive eigenvalue $\sigma _3$.

**Fig. 11:**
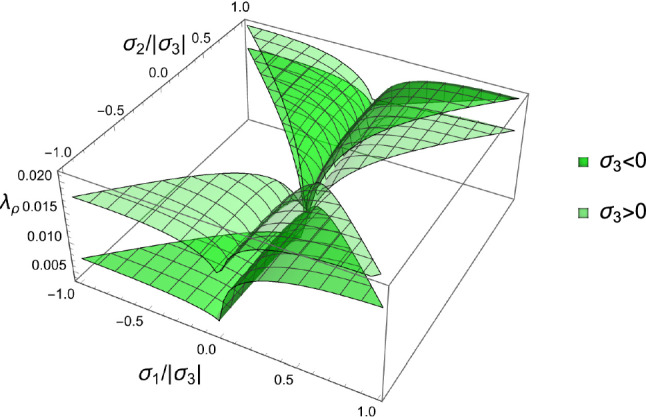
The relative damage function $y/y_{set}$ is the factor $f(\rho )\lambda _S$ and is shown as a function of the principal stress ratios for the optimal fabric ratios. The minimum damage is achieved in hydrostatic compression

As shown in Fig. 11, the minimum density over stress intensity ratio is achieved for stress states belonging to the quadrant containing hydrostatic compression.

#### The 2D case

In case $\hat{\sigma }_1=0$, the fabric tensor $\textbf{M}$ is of rank 2 and Eq. S.28 reduce to 3 scalar equations for 2 fabric eigenvalues and the multiplier $\lambda _y$:

29 $$\begin{aligned} \frac{2F_0^2}{\sqrt{\hat{\textbf{S}}:\mathbb {F}\hat{\textbf{S}}}}(\zeta _0 (\sum _{k=2}^3 \frac{\hat{\sigma }_k}{m_k^2}) \frac{\hat{\sigma }_i}{m_i} -(1+\zeta _0) \frac{\hat{\sigma }_i^2}{m_i^3}) - 2f_0 \frac{\hat{\sigma }_i}{m_i} - \lambda _y m_i^2= & 0 \;\; i=2,3 \nonumber  \sum _{k=2}^3 m_k= & 2 \end{aligned}$$

**Fig. 12:**
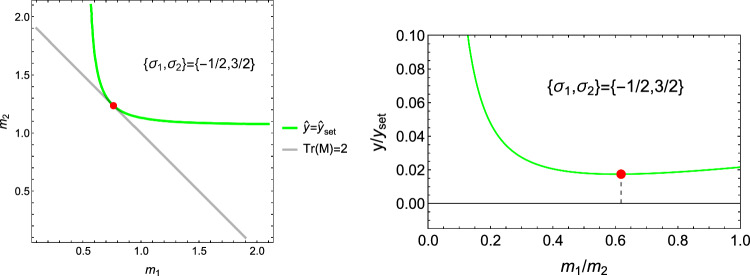
Left: the optimal fabric ratio for a given stress ratio. Right: the yield function against fabric ratio with the corresponding minimum

**Fig. 13:**
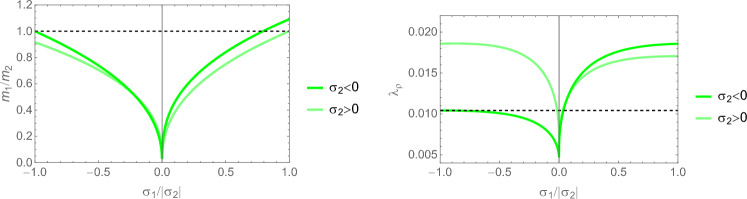
Left: the optimal fabric ratio for a given stress ratio. Right: the density-to-stress intensity ratio for a negative $\sigma _2<0$ and positive $\sigma _2> 0$ eigenvalue along $^2\textbf{e}$

A particular solution is shown in Fig. 12 and the general solutions are plotted in Fig. 13.

#### The 1D case

This is the case $\hat{\sigma }_1=0 \quad \hat{\sigma }_2=0$ and $\textbf{M}={^3\textbf{e}} \otimes {^3\textbf{e}}$. The yield function becomes

30 $$\begin{aligned} y(\hat{\sigma }_3)=\hat{\sigma }_3 \frac{1}{2}(\frac{1}{\sigma _0^+}-\frac{1}{\sigma _0^-})+ |\hat{\sigma }_3|\frac{1}{2}(\frac{1}{\sigma _0^+}+\frac{1}{\sigma _0^-})=y_{set}\frac{f(\rho )}{\lambda _S} \end{aligned}$$

**Fig. 14:**
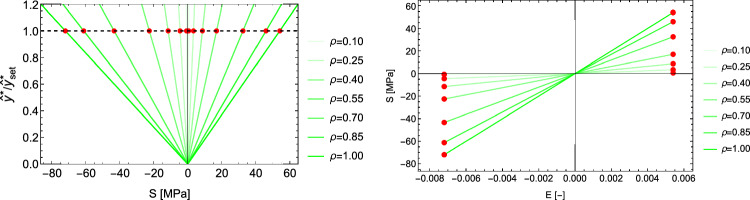
Left: in 1D, the yield criterion is an asymmetric absolute value function of stress, that reaches the highest stresses for $\rho =1$. Right: in the stress–strain diagram, the criterion exhibits constant but distinct homeostatic strains in compression and tension. The red dots indicate the set-points in damage on the left and expressed in strains on the right

An elastic modulus of 10 GPa, the selection $y_{set}=1$, yield stresses in compression and tension of $\sigma _0^+=54$ MPa and $\sigma _0^-=72$ MPa, lead to constant yield strains of 0.0054 in tension and 0.0072 in compression, respectively. As desired, the 1D case degenerates into a mechanostat with distinct thresholds in tension and compression (Fig. 14).

### Principal strains

#### Formulation

A third model inspired by the underlying fluid flow mechanism consists in assuming that the stimulus is not a scalar, but that all principal material orientations are strained to reference amplitudes that can differ in tension and compression. Accordingly, the stimulus becomes a strain tensor $\textbf{E}_{set}$ that is diagonal in the fabric coordinate system and the principal stresses are aligned with the fabric tensor due to the orthotropic nature of the fabric–elasticity relationships.

31 $$\begin{aligned} \textbf{E}(\textbf{S}) = \mathbb {E}(\rho ,\textbf{M}) \textbf{S}={^{set}\textbf{E}}=\sum _k {^{set}E}_{k}({^k\textbf{s}} \otimes {^k\textbf{s}}) \end{aligned}$$

with ${^k\textbf{s}}$ the normed stress eigenvectors of $\textbf{S}$, and ${^{set}E}_{k}=\pm {^{set}E}_{\pm }$ the reference strain levels in tension or compression for each direction k.

In this formulation, there is no minimisation, the alignment between principal stresses and strains is prescribed *a priori*, and fabric eigenvalues are sought to achieve admitted strain amplitudes along each principal direction. These set-points can either be a symmetric stimuli corresponding to fluid-flow homeostasis ($E^{-}_{set}=E^{+}_{set}$) or an asymmetric maximal stimuli corresponding to a damage level along each principal stress direction. For each stress tensor, a density and fabric is sought that leads to appropriate strain states among the 8 corners of the box shown in Fig. S3. The resolution and discussion of the existence/unicity of the solution are provided in subsection 3.3 of supplementary material.

#### Solution

**Fig. 15:**
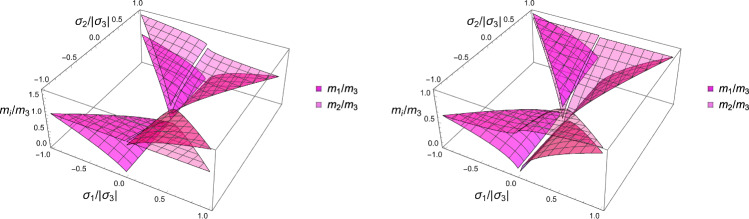
The fabric eigenvalue ratios as a function of the principal stress ratios for a negative (left) and positive (right) principal stress along direction 3. Similarly to the damage criterion there is a distinct solution in compression and tension

The solution for the fabric ratios is shown for a negative and a positive stress eigenvalue along the axis ${^3\textbf{e}}$ in Fig. 15.

**Fig. 16:**
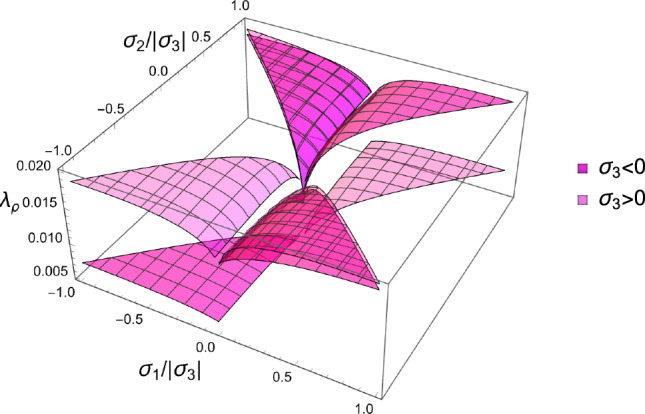
The factor $\lambda _{\rho }=f(\rho )/\lambda _S$ exhibits two surfaces depending on the sign of the third stress eigenvalue $\sigma _3$. In general, the lowest values are achieved in the hydrostatic compression quadrant where all stress and strain eigenvalues are negative

The corresponding solution for the density-to-stress intensity ratio $\lambda _{\rho }$ is shown in Fig. 16.

#### The 2D case

**Fig. 17:**
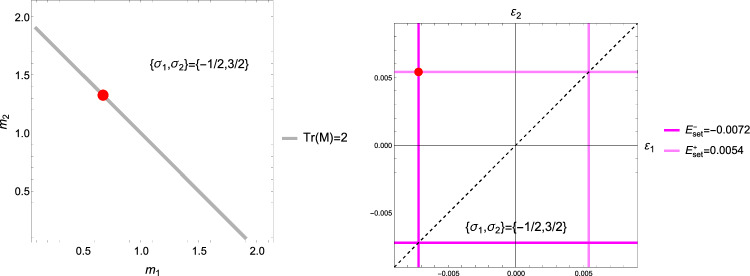
Left: a particular solution for a given stress ratio in fabric space. Right: the same solution in strain space

**Fig. 18:**
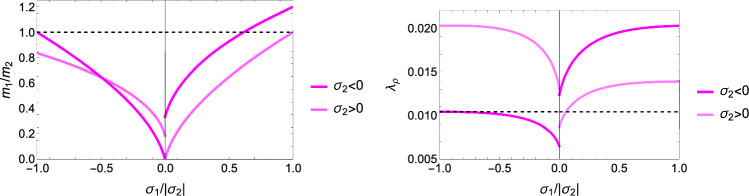
Left: the optimal fabric ratio for a given stress ratio. The ratios are discontinuous at zero due to the different strain set-points in tension and compression. Right: the $\lambda _{\rho }$ parameter with respect to the principal stress ratios. The dashed line is the value achieved for hydrostatic stress

In case $\sigma _1=\hat{\sigma _1}=0$, the fabric tensor $\textbf{M}$ is of rank 2 and Eq. S.36 reduces to 3 scalar equations for 2 fabric eigenvalues and the multiplier $\lambda _{\rho }$ (Figs. 17 and 18):

32 $$\begin{aligned} -\frac{\nu }{\epsilon } (\sum _{k=2}^3 \frac{\hat{\sigma }_k}{m_k}) \frac{1}{m_i} +\frac{(1+\nu )}{\epsilon } \frac{\hat{\sigma }_i}{m_i^2} - \lambda _{\rho } (-\mathcal {H}(-\hat{\sigma }_i)E^{-}_{set}+\mathcal {H}(\hat{\sigma }_i) E^{+}_{set})= & 0 \nonumber \\ i= & 2,3 \nonumber \\ \sum _{k=2}^3 m_k= & 2 \end{aligned}$$

#### The 1D case

This is the case $\sigma _1=0 \quad \sigma _2=0$ and $\textbf{M}={^3\textbf{e}} \otimes {^3\textbf{e}}$. The homeostatic strains are the ones prescribed for each principal strain axis, that is 0.0054 in tension and $-0.0072$ in compression. For density we simply have

33 $$\begin{aligned} \rho = f^{-1}(\frac{S}{\epsilon _0 {^{set}E}}) \end{aligned}$$

**Fig. 19:**
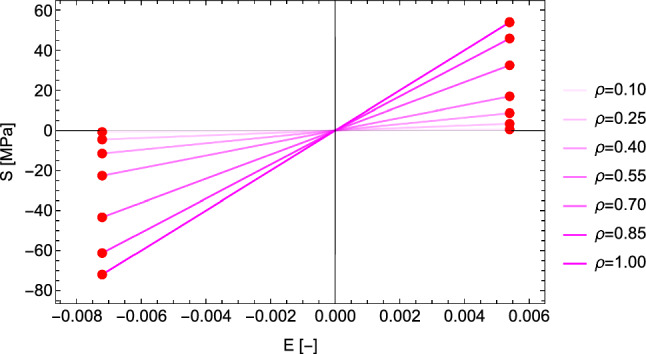
Stress-strain diagram where the red dots indicate distinct homeostatic strains in compression and in tension that are independent of density

As shown in Fig. 19, the 1D case of the principal strain metric coincides precisely with the one of the GYC.

### Comparison

**Fig. 20:**
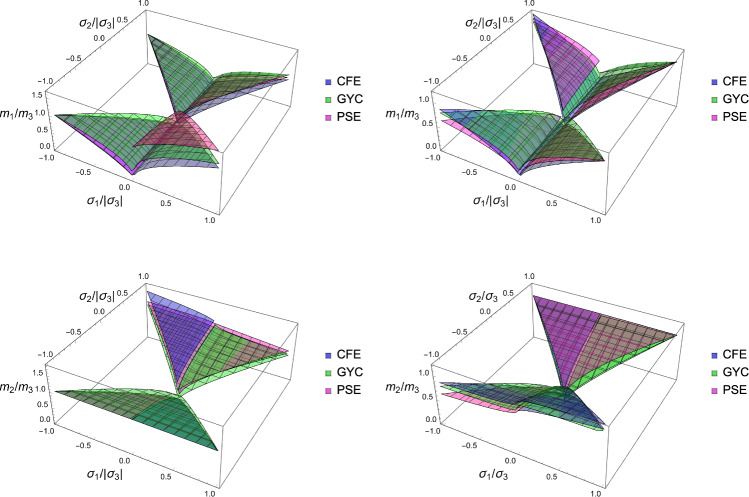
Comparison of the fabric ratios $m_1/m_3$ (top) and $m_2/m_3$ (bottom) for the negative (left), respectively, positive (right) stress eigenvalue, and for the three mechanostats CFE, GYC and PSE

**Fig. 21:**
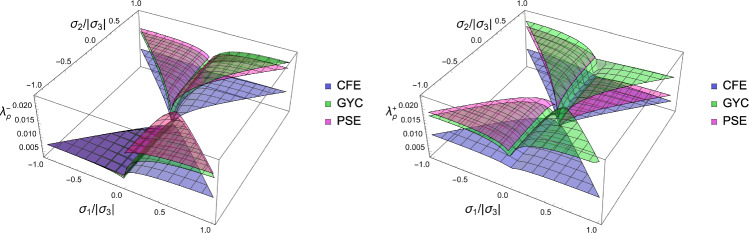
Comparison of $\lambda _{\rho }$ for the negative (left), respectively, positive (right) third stress eigenvalue for the three mechanostats CFE, GYC and PSE

**Fig. 22:**
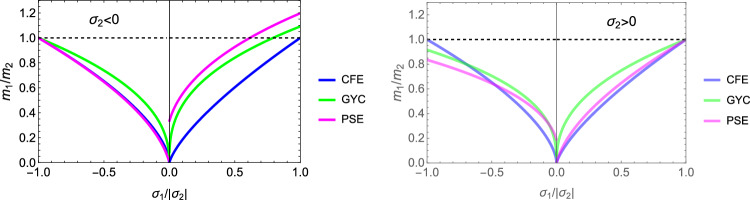
Comparison of the fabric ratios $m_2/m_3$ for the negative (left), respectively, positive (right) stress eigenvalue, and for the three mechanostats CFE, GYC and PSE

**Fig. 23:**
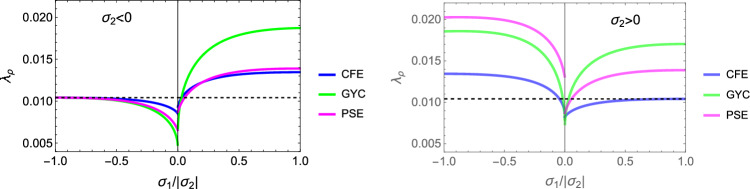
Comparison of the density-to-stress ratios $\lambda_{\rho}$ for the negative (left), respectively, positive (right) stress eigenvalue, and for the three mechanostats CFE, GYC and PSE

In this section, the obtained solutions for the three considered set-points: normalised complementary free energy density (CFE), the generalised yield criterion (GYC) and the principal strain (PSE) are compared in both 3D and 2D. Figure 20 shows the solution for the fabric ratios in 3D. These are rather similar in the negative and positive quadrants of the stress ratios. Stronger singularities are visible for the PSE set-point. Figure 21 compares the $\lambda _{\rho }$ parameter that stands for the ratio of density over stress intensity. The lowest ratio is achieved by GYC in the negative and by CFE in the positive quadrant of the principal stress ratios. In 2D, the fabric eigenvalue ratios are shown in Fig. 22 and the $\lambda _{\rho }$ factor in Fig. 23. Again, the solutions for the ratios are comparable in the negative and positive quadrants and the minimum $\lambda _{\rho }$ is obtained for the PSE set-point.

For a given stress state, the three set-points $\overline{\psi ^*}_{set}$, $y_{set}$, and $\textbf{E}_{set}$ described above lead to qualitatively similar but quantitatively different optimal fabric tensors. Importantly, the three formulations lead to the alignment of the fabric with the stress tensor. In general, the fabric eigenvalue ratios increase with the corresponding principal stress ratios and are less sensitive to the other stress ratio.

In contrast to the complementary free energy density, the damage and principal strain set-points lead to distinct optimal fabric tensors for an opposed stress $-\textbf{S}$.

An interesting feature of the principal strain set-point $\textbf{E}_{set}$ is that it can be directly specialised to a 2D or even 1D cases where one or two principal stress components vanish. The 3D framework would predict that the corresponding fabric eigenvalue becomes zero. A 2D case could be applied to a compact bone shell where the normal stress is essentially zero and a 2D fabric tensor is representing the lamellar orientations (Franzoso and Zysset 2009). Similarly to the 3D case, the fabric tensor would align with the principal axes of the orientation distribution function of the MCFs. The aspect ratio would derive from the anisotropic elastic properties of the lamellar plywood structure.

The CFE and GYC criteria are expressed in the functional stress space to address the stated problems but can be transformed in strain space using the fabric-based linear elasticity relationship between stress and strain. Note that due to proper normalisation, all criteria are macroscopic strain metrics that are independent of density.

The 1D case corresponds to a single trabecula that is subjected exclusively to axial stresses. The stress should simply align with the trabecula, and the strain set-point would be associated with the change of volume that is responsible for the poroelastic drag of bone fluid in the lacuno-canalicular network contributing to mechanosensation. The difference in the tensile and compressive strain set-points emerges from the different yield levels in the two loading modes.

## Inverse problem

As stated in the introduction, the inverse problem consists in finding an optimal stress tensor for a given bone density and fabric tensor. In the same spirit, optimality is understood as bearing the highest stress intensity for a given bone volume fraction and is sought among all the possible stress states of equivalent intensity defined by $\textrm{tr}|\textbf{S}|$. The stress intensity and tensorial orientation are therefore again uncoupled, and a minimisation is looked for among the different stress states. For a given mechanostat, the stress intensity is then derived for the retained tensorial orientation.

The 3 same metrics will be considered, the normalised complementary free energy density (CFE), the generalised yield criterion (GYC), and the principal strains (PSE) for an identical set-point that corresponds to an isotropic contractile strain of 0.72 %.

### Normalised complementary free energy density (CFE)

The CFE is considered with the same normalisation and set-point as in Eq. 14

$$\begin{aligned} \widehat{\psi ^*}(\textbf{S};\rho ,\textbf{M})= \frac{1}{2} \frac{f(\rho )}{\hat{f}^2(\rho )}\textbf{S}:\mathbb {E}(\rho ,\textbf{M}) \textbf{S}=\widehat{\psi ^*_{set}} \end{aligned}$$

#### Formulation

As established in the forward formulation, using $\textbf{S}=\lambda _{S}(\textbf{M})\hat{\textbf{S}}$, and $\mathbb {E}(1,\textbf{M})=f(\rho )\mathbb {E}(\rho ,\textbf{M})$ we have

34 $$\begin{aligned} \widehat{\psi ^*}(\hat{\textbf{S}};1,\textbf{M})=\widehat{\psi ^*_{set}} \left( \frac{\hat{f}(\rho )}{\lambda _S(\textbf{M})}\right) ^2=\widehat{\psi ^*_{set}}\lambda _{\rho }^2(\rho ,\textbf{M}) \end{aligned}$$

In contrast to the forward formulation, we are looking for the optimal density-to-stress intensity ratio by minimising the CFE with respect to the stress tensor under a stress normalisation constraint:

35 $$\begin{aligned} \overline{\hat{\textbf{S}}} = \textrm{Arg Min}_{\{\hat{\textbf{S}},\textrm{tr}|\hat{\textbf{S}}|=3\}} \; \widehat{\psi ^*}(\hat{\textbf{S}};1,\textbf{M}) \end{aligned}$$

For a given fabric, the outcome of the minimisation delivers the direction of the stress tensor $\hat{\textbf{S}}$. The given density delivers then the optimal stress multiplier

36 $$\begin{aligned} \overline{\lambda _S}(\rho ,\textbf{M})=\frac{\hat{f}(\rho )}{\overline{\lambda _{\rho }}(\textbf{M})} \qquad \overline{\lambda _{\rho }}(\textbf{M})=\sqrt{\frac{\widehat{\psi ^*}(\overline{\hat{\textbf{S}}};1,\textbf{M})}{\widehat{\psi ^*_{set}}}} \end{aligned}$$

**Fig. 24:**
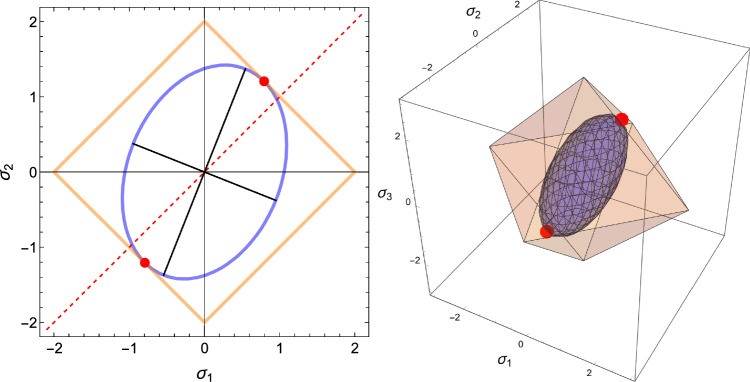
The elliptical/ellipsoidal surface of the CFE is shown in 2D/3D together with the one of the normalisation constraints of the stress tensor that takes the form of two opposed pyramids/triangles. For a given fabric, the sought minimum is the first intersection between the two surfaces when increasing the stress intensity. Since the CFE and the constraint surface are invariant to stress tensor sign, the minimum is achieved on two symmetric points

The resolution of the problem and discussion of existence/unicity are provided in subsection 4.1 of supplementary material (Fig. 24).

#### Solution

**Fig. 25:**
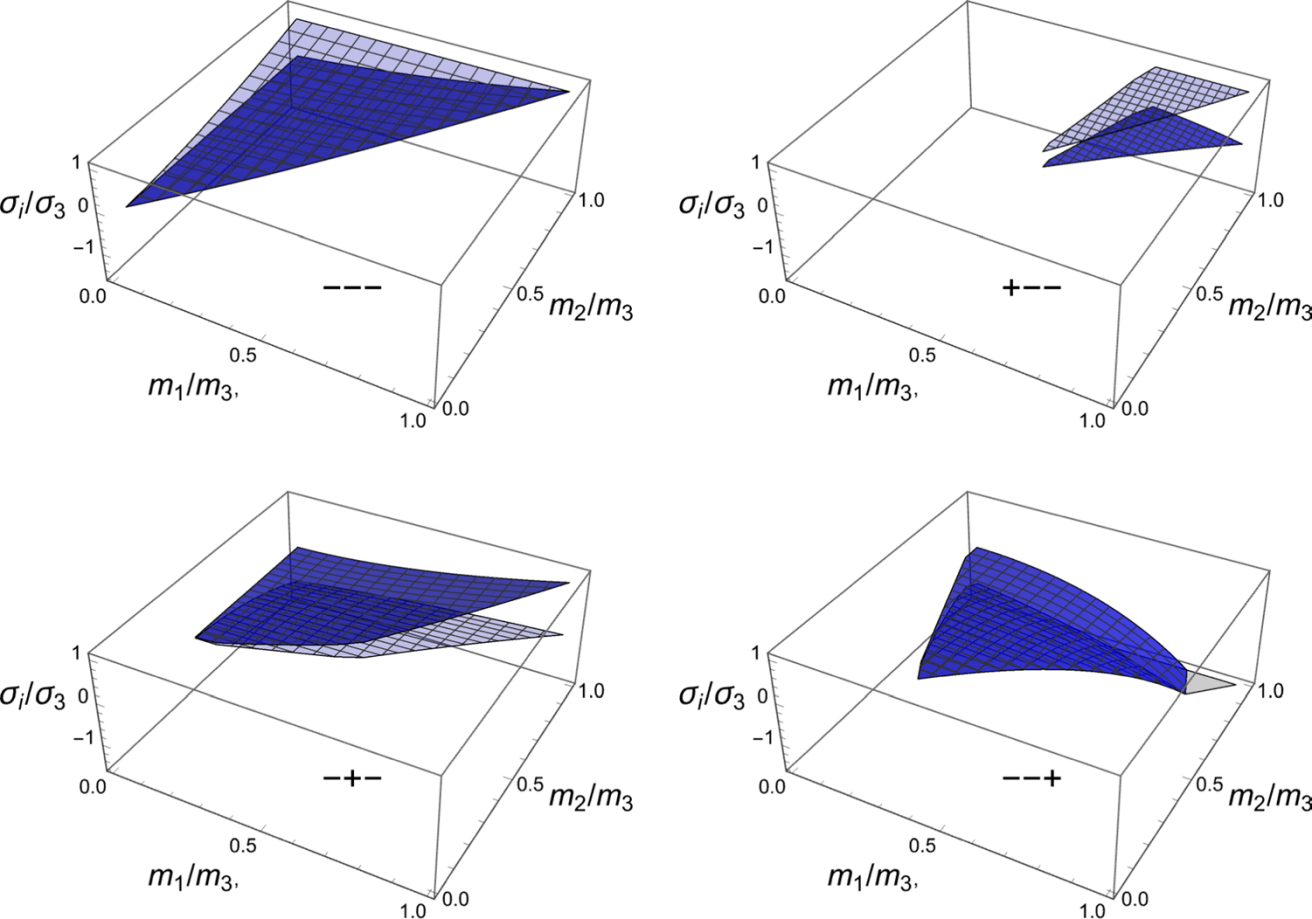
The stress ratios $\sigma _1/\sigma _3$ (dark) and $\sigma _2/\sigma _3$ (light) that lead to the minimum CFE for the full domain of fabric ratios. Due to symmetry, the solution of the opposed octants is identical

The stress ratios that satisfy the stationary equations are shown in Fig. 25 for each octant.

**Fig. 26:**
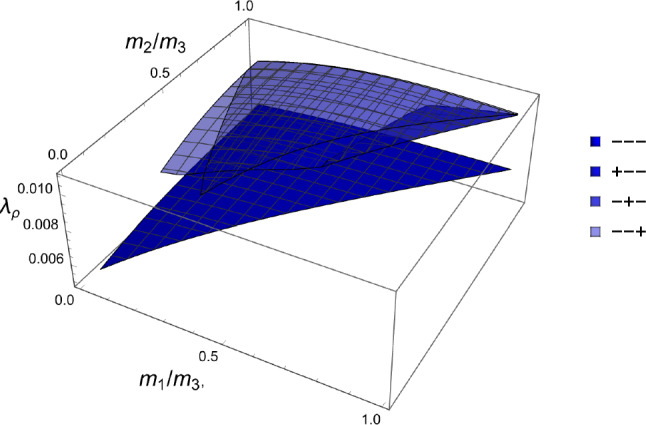
Density-to-stress intensity ratio $\lambda _{\rho }$ corresponding to the minimum CFE for the full domain of fabric ratios and the four distinct octants. The surface corresponding to the fully negative (or positive) principal stresses is the lowest over the entire fabric domain

The resulting density-to-stress intensity ratio of these different solutions for the stress ratios, corresponding to the minimum CFE, is shown in Fig. 26. The minimum surface corresponding to the fully negative (or positive) octant is the lowest over the entire fabric domain, which indicates that stress states with principal stresses of the same sign lead to a lower density with respect to a given stress intensity.

#### 2D case

Specialisation of Eq. 42 to 2D gives

37 $$\begin{aligned} \hat{\sigma }_i= & \lambda _{\psi } (\lambda _0 (\sum _{l=2}^{3} m_l \frac{\hat{\sigma }_l}{|\hat{\sigma _l}|}) m_i + 2\mu _0 \frac{\hat{\sigma }_i}{|\hat{\sigma }_i|} m_i^2) \;\; i = 2,3 \nonumber \sum _{l=2}^3 |\hat{\sigma }_l|= & 2 \end{aligned}$$

**Fig. 27:**
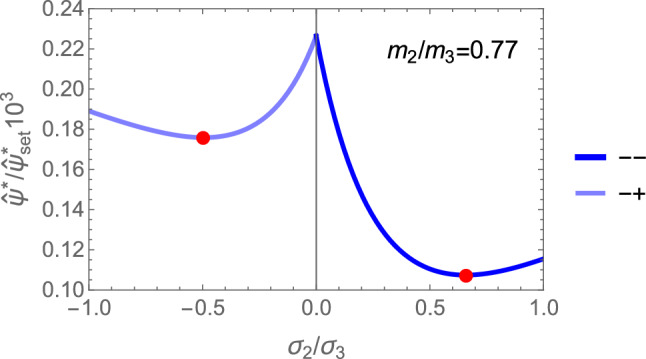
Example of a 2D CFE function and its minima for a representative value of $m_2$/$m_3$. In this case, a stationary point exists in both quadrants, but the corresponding CFE value is lower when the sign of the stress eigenvalues is the same

An example of the 2D CFE is shown for a given fabric in Fig. 27.

**Fig. 28:**
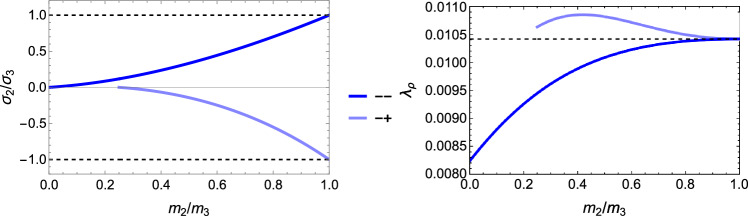
Comparison of the stress ratio $\sigma _2/\sigma _3$ for the two distinct quadrant. In the hybrid quadrant q_+−_, there is no solution for $m_2/m_3=1< 1/3$. The minimum is achieved in the positive quadrant for any fabric ratio. The values of -1 and +1 are recovered for $m_2/m_3=1$

The solution for the stress ratios and the amplitude $\lambda _{\rho }$ is provided in Fig. 28.

#### 1D case

In the uniaxial case, the set-point of the normalised free energy density $\Psi _{set}$ corresponds to a stress of 72 MPa or a strain of 0.72 % related by the elastic modulus of 10 GPa.

### Generalised yield criterion (GYC)

Taking the same approach we investigate the generalised yield criterion that is expressed in stress due to the frequent displacement-based formulation of finite element analysis. However, the criterion in the stress space can always be mapped into the strain space over the linear but anisotropic elasticity.

#### Formulation

The generalised yield criterion writes

38 $$\begin{aligned} y(\textbf{S};\rho , \textbf{M})=\sqrt{\textbf{S}:\mathbb {F}(\rho ,\textbf{M})\textbf{S}}+\textbf{F}(\rho ,\textbf{M}):\textbf{S}=y_{set} \end{aligned}$$

**Fig. 29:**
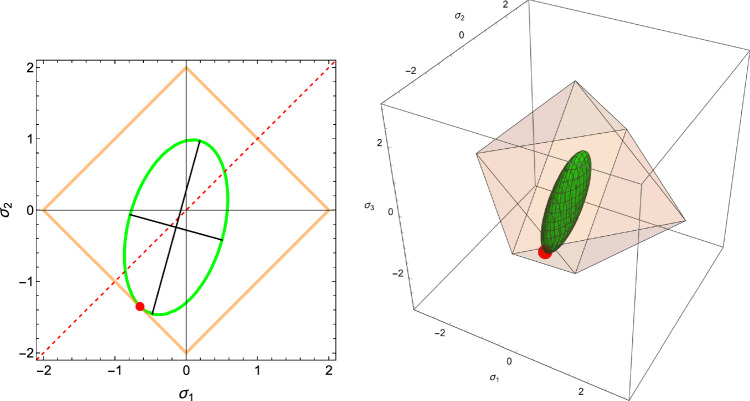
The sought minima in 2D/3D appears at the first intersection between the yield criterion and the stress normalisation constraint. Unlike the CFE, and due to the shift of the quadratic yield form with respect to the origin, a solution appears first in compression

Homogeneity of degree one with respect to both stress and bone density implies that

39 $$\begin{aligned} y(\hat{\textbf{S}};1,\textbf{M})=y_{set}\frac{\hat{f}(\rho )}{\lambda _S}=y_{set} \lambda _{\rho } \end{aligned}$$

with $y_{set}>0$ representing different levels of yield or damage. Following the forward formulation, we are looking for the optimal density-to-stress intensity ratio by minimising the GYC with respect to the stress tensor under the normalisation constraint (Fig. 29):

40 $$\begin{aligned} \overline{\hat{\textbf{S}}} = \textrm{Arg Min}_{\{\textrm{tr}|\hat{\textbf{S}}|=3\}} \; y(\hat{\textbf{S}};1,\textbf{M}) \end{aligned}$$

For a given density, the stress multiplier is given by

41 $$\begin{aligned} \lambda _{S}(\rho ,\textbf{M})=\frac{\hat{f}(\rho )}{\lambda _{\rho }(\textbf{M})} \qquad \lambda _{\rho }(\textbf{M})=\frac{y(\overline{\hat{\textbf{S}}};1,\textbf{M})}{y_{set}} \end{aligned}$$

The resolution of the problem and the discussion of existence/unicity are presented in subsection 4.2 of the supplementary material.

#### Solution

**Fig. 30:**
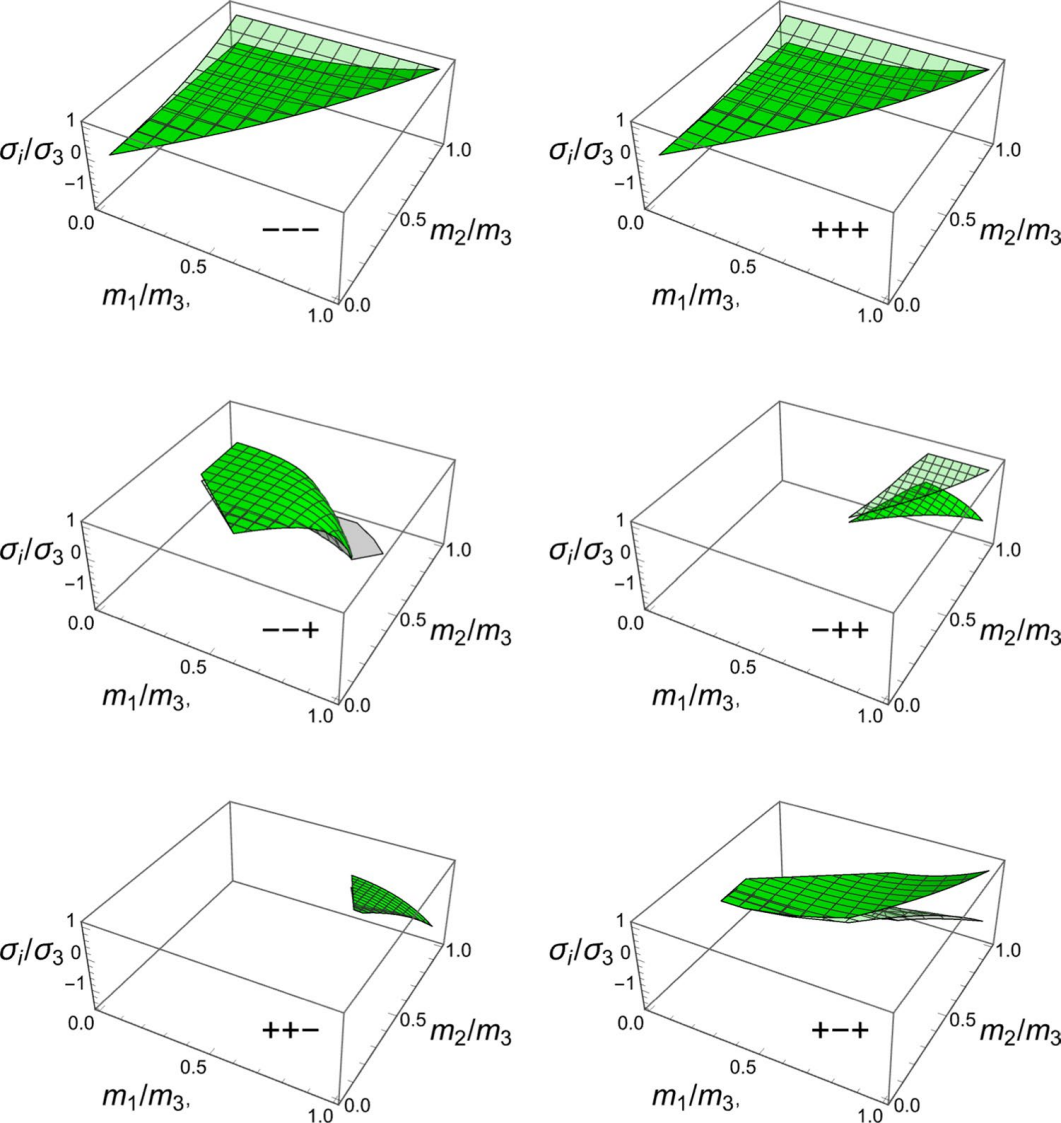
The stress eigenvalue ratios $\sigma _i/\sigma _3$ for the minimum of the GYC criterion in the full domain of the fabric eigenvalue ratios and for the six octants that exhibited some solutions. The octants with the same sign for all stress eigenvalues have solutions for the full domain of the fabric eigenvalues

The stress ratios that achieve a minimum are shown in Fig. 30 for the octants that exhibited some solutions.

**Fig. 31:**
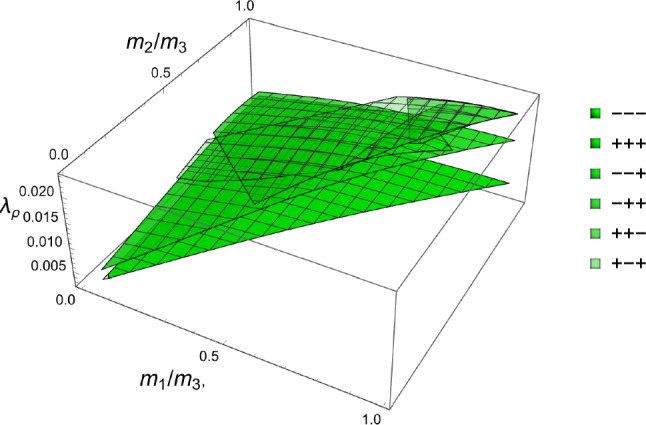
Density-to-stress intensity ratio $\lambda _{\rho }$ for the minimum GYC criterion in the full domain of the fabric eigenvalue ratios. Among the existing solutions, the one with the three negative stress eigenvalue has the lowest intensity for any fabric

The scaling ratios of the solutions are plotted in Fig. 31

#### 2D case

Specialisation of Eq. 42 to 2D gives

42 $$\begin{aligned} \hat{\sigma }_i= & \lambda _y (\lambda _0 (\sum _{l=2}^{3} m_l \frac{\hat{\sigma }_l}{|\hat{\sigma _l}|}) m_i + 2\mu _0 \frac{\hat{\sigma }_i}{|\hat{\sigma }_i|} m_i^2) \nonumber \;\; i= & 2,3 \nonumber \;\; \sum _{l=2}^3 |\hat{\sigma }_l|= & 2 \end{aligned}$$

**Fig. 32:**
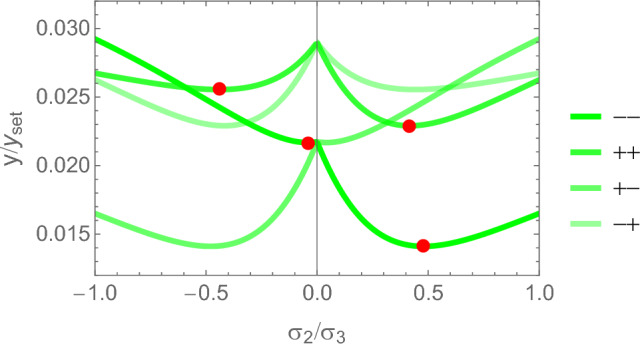
The relative 2D yield criteria with the different minima in their respective quadrants for a representative fabric ratio of 0.77

An example of the minima of the GYC for a representative ratio of fabric eigenvalues is shown in Fig. 32.

**Fig. 33:**
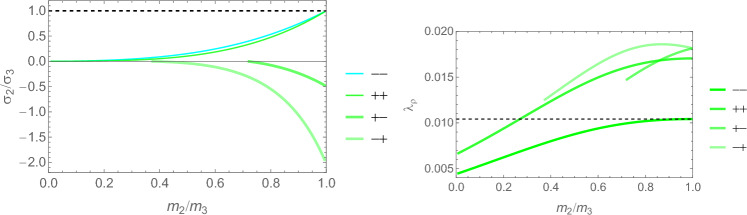
Comparison of the stress ratios $\sigma _2/\sigma _3$ for the 4 distinct quadrants. The isotropic values of +1 are recovered for $m_2/m_3=1$ when the signs of the two stress eigenvalues are identical, but the ratios are not exactly identical. Solutions are missing for lower values of the fabric ratios

The solution for the stress ratios and the amplitude $\lambda _{\rho }$ is provided in Fig. 33.

#### 1D case

In the uniaxial case, the set-point of the normalised free energy density $\hat{y}_{set}$ corresponds to a minimum uniaxial stress of -74 MPa.

### Principal strains

**Fig. 34:**
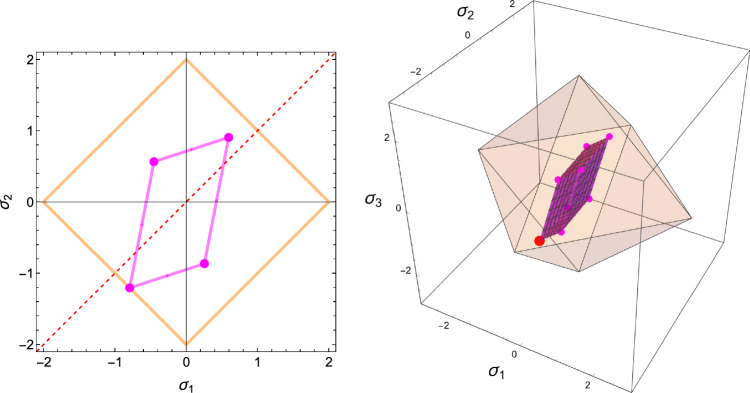
The stress domain resulting from the cubic strain domain with the same stress norm surface used for the CFE and GYC metric. The stress state produced by three negative strain eigenvalues reaches the norm surface first by increasing the stress intensity

We are now looking for the stress tensors that reflect a homeostatic principal strain level for a given density and fabric tensor $\textbf{M}$. The admissible strain states belong to the corners of an off-centre cubic domain that correspond to combinations of two distinct strain levels $\{-E^{-}_{set},E^{+}_{set}\}$ in compression and tension, respectively. The off-centre cubic strain domain is transformed into a hexahedral domain in stress space with corresponding corners (Fig. 34). The inverse problem consists therefore in finding the stress states that minimise the density-to-stress intensity ratios with the benefit that explicit stress states are delivered by the density- and fabric-based Hooke’s law.

43 $$\begin{aligned} \hat{\textbf{S}}=\frac{1}{\lambda _S}\textbf{S} = \frac{1}{\lambda _S}\mathbb {S}(\rho ,\textbf{M}){^{set}\textbf{E}} = \frac{f(\rho )}{\lambda _S}\mathbb {S}(1,\textbf{M}){^{set}\textbf{E}} \end{aligned}$$

Based on the 8 admissible strain states, a list of 8 normalised stresses are therefore candidate for the sought stress state.

The density-to-stress intensity ratio can then be calculated for each density and fabric

44 $$\begin{aligned} \lambda _{\rho } = \frac{f(\rho )}{\lambda _S} \qquad \textrm{with} \qquad \lambda _S=\frac{tr|\textbf{S}|}{3} \end{aligned}$$

In the case of the principal strain box with the selected (realistic) yield strains, and in contrast with the previous criteria, an explicit solution exists in each principal stress octant for any fabric. A global minimum for the density function over stress intensity ratio is found in the hydrostatic octant for any fabric.

#### Solution

**Fig. 35:**
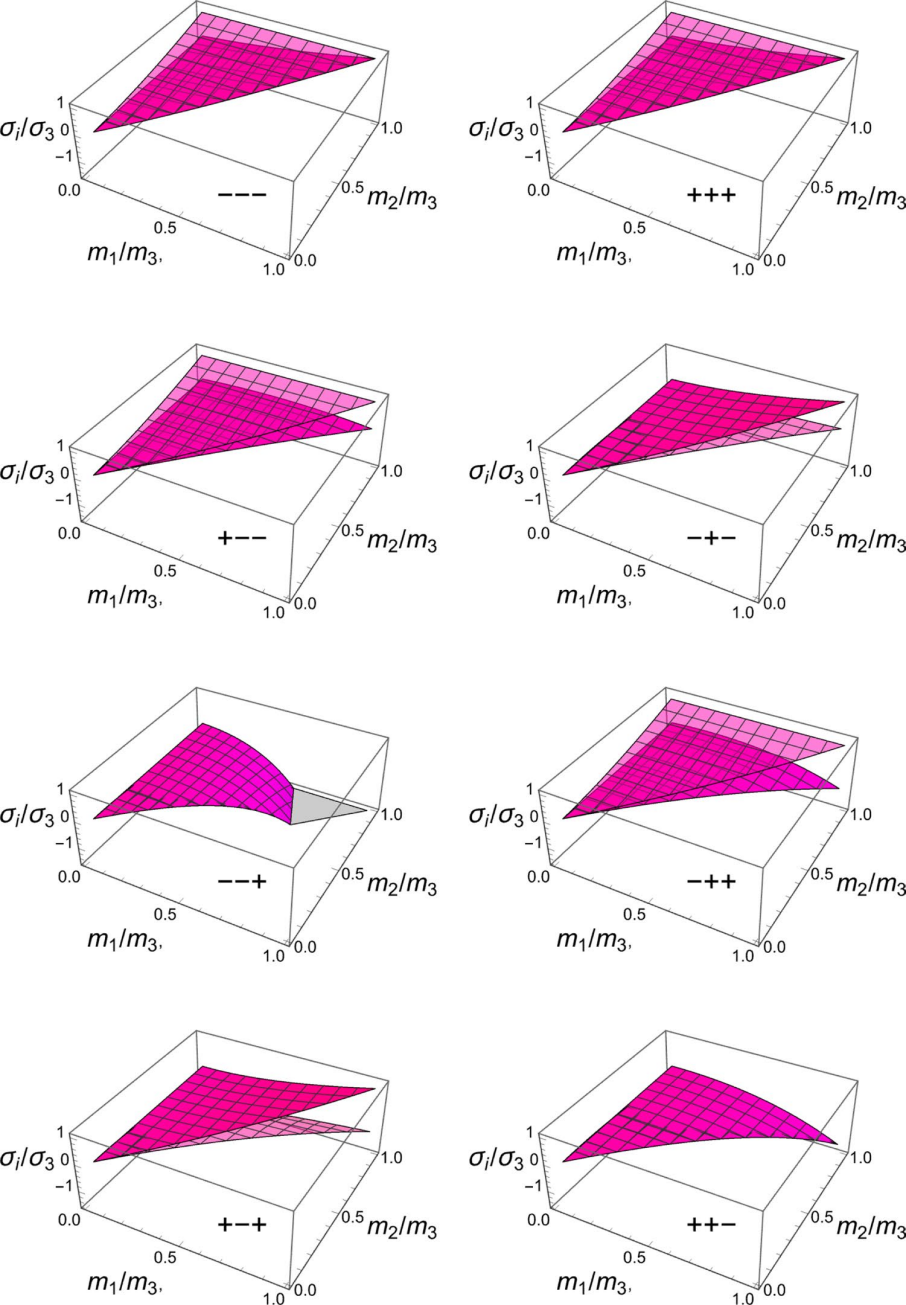
The principal stress ratios as a function of the fabric ratios for all octants

The principal stress ratios obtained for each strain state are shown for the full fabric domain in Fig. 35. Given the explicit formulation of stress as a function of density and fabric, a full set of solutions is obtained in each octant. Like for CFE, the results of octant $\mathrm{o}_{---}$ and $\mathrm{o}_{+++}$ are identical due to the same elastic anisotropy and strain ratios in compression and tension.

**Fig. 36:**
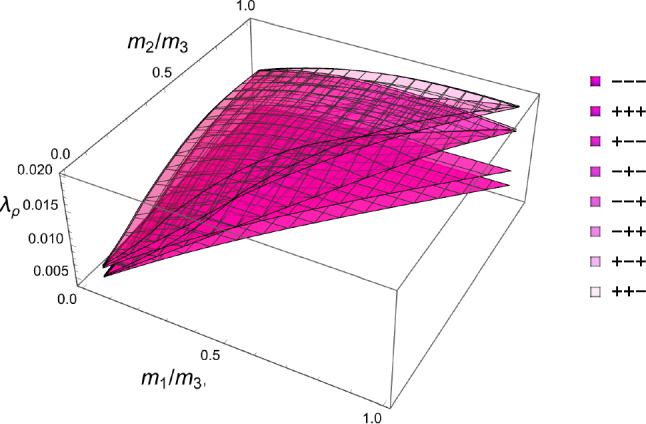
The lambda parameter for the 8 strain octants. The negative octant $\mathrm{o}_{---}$ provides the lowest density-to-stress intensity ratio for any fabric

The corresponding factors $\lambda _{\rho }$ are shown in Fig. 36. Similar to what was found in the forward problem, the fully negative octant $\mathrm{o}_{---}$ corresponding to a hydrostatic but usually not isotropic pressure, leads to the lowest bone density-to-intensity ratio for any fabric.

#### 2D

**Fig. 37:**
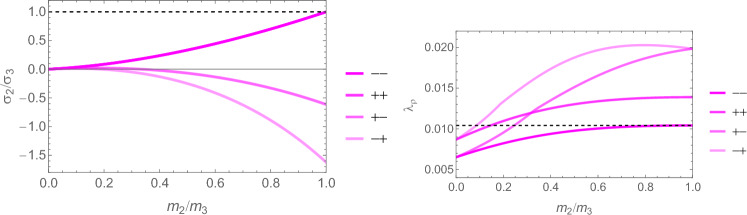
The principal stress ratios (left) and the $\lambda _{\rho }$ parameter as a function of the fabric ratios for the 4 quadrants

Following the same formulation, the explicit solutions for the fabric ratios and $\lambda _{\rho }$ factor are shown in Fig. 37. Again, the fabric ratios are equivalent for the $q_{--}$ and $q_{++}$ quadrant.

#### 1D

The 1D case degenerates into the uniaxial stress $\sigma _3=72$ MPa with $\lambda _{\rho }=1/72$.

### Comparison of the three criteria

**Fig. 38:**
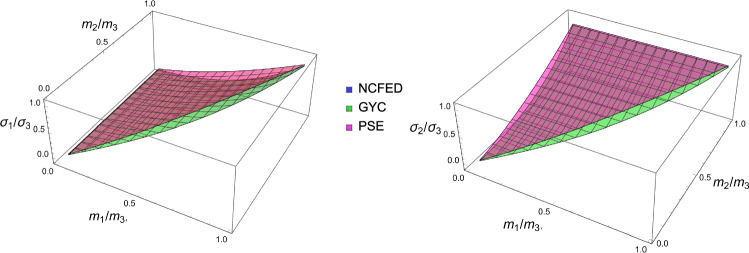
Comparison of the stress ratios for the CFE, GYC and PSE criteria. The CFE and PSE stress ratios are identical as they are based on the same anisotropic elasticity tensor

**Fig. 39:**
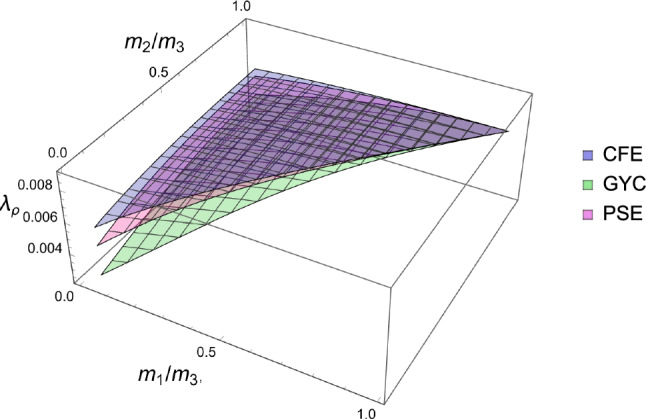
Comparison of the density-to-stress intensity ratio for the CFE, GYC and PSE criteria. The amplitudes converge correctly in the isotropic corner (1.0, 1.0) as the same hydrostatic strain state was used for the three criteria

For the 3D case, the comparison of the fabric ratios is shown in Fig. 38 and the density-to-stress intensity ratio in Fig. 39

**Fig. 40:**
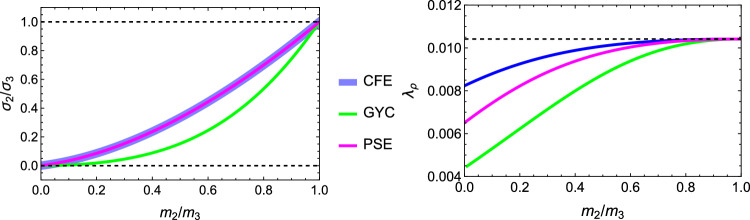
Comparison of the stress ratios and the density-to-stress intensity ratios for the CFE, GYC and PSE criteria. Again, the stress ratios are identical for CFE and PSE. The amplitudes converge again correctly for 1.0

For the 2D case, the comparisons of both the fabric ratios and the $\lambda _{\rho }$ factor of the negative $\mathrm{q}_{--}$ quadrant are shown in Fig. 40.

## Discussion

### Summary

In the perspective of efficient computation of forward and inverse bone adaptation problems on clinical images, the aim of this study was to formulate and find the solutions of optimisation problems based on several mechanostat criteria at the RVE level by exploiting fabric–elasticity and fabric–yield relationships. Three convex strain metrics, the normalised complementary free energy (CFE), a yield/damage metric (GYC) and a principal strain box (PSE) were investigated in both the forward and the inverse problem. Existence and unicity of the solutions were examined. All solutions were reported in 3D, specialised to 2D and 1D and compared among the three convex metrics.

### Forward problem

As reported by Luo and An (1998), the spectral decomposition of the fabric tensor must align with the one of the stress tensor to minimise the CFE, and this important result was extended in this work to the asymmetric GYC criterion. In the present work, we extended their preliminary results on the forward CFE problem with explicit solutions for the fabric and stress eigenvalues.

Since no general analytical solutions were available, a numerical scheme was applied to resolve the derived scalar equations for specific material parameters of the fabric–elasticity or fabric–yield relationships. For CFE, the obtained relationships between stress and fabric eigenvalue ratios as well as stress intensity to density ratio depend on Poisson’s ratio but neither on Young’s modulus nor on the shear modulus. For GYC, the solutions depend on the ratio of the tensile versus compressive uniaxial yield stresses $\sigma _0^+/\sigma _0^-$ as well as the shape factor $\zeta _0$ but not on the shear yield stress. For the PSE criterion, the alignment of principal strains and fabric is assumed and the solution depends again on Poisson’s ratio and the differences between tensile and compressive set-point strains but not on Young’s or shear modulus. This is essentially due to the fact that fabric, the principal strains and stresses are aligned in all cases, which means that no shear stresses appear in the material coordinate system and Young’s modulus is a simple scaling factor of the principal stresses (see Fig. 1).

Existence and unicity of the forward solutions relies on convexity of the selected criteria which was anticipated in Fyhrie and Carter (1986). This means that for any applied stress tensor on a trabecular RVE, a unique fabric tensor minimises bone density for the selected mechanostat at the defined set-point. However, the derived solutions become singular when a fabric or stress eigenvalue tends to zero. Considering trabecular bone, the measured fabric eigenvalues are much larger than zero and this restriction has no practical consequences. On the other hand, when a principal stress tends towards zero, a solution can be retrieved for the nonzero principal stresses from the problem projected at a lower dimension. The 1D case was included to make the link with the original mechanostat by Frost that applies to a scalar set-point uniaxial strain value (Frost 2003).

Interestingly, the lowest density-to-stress intensity ratio $\lambda _{\rho }$ is found in the negative hydrostatic pressure quadrant independently of the selected criterion in 2D or 3D. For symmetric criteria, the negative and positive hydrostatic quadrants become equivalent. Moreover, within the negative hydrostatic pressure quadrant, the lowest density-to-stress intensity ratio is found when one of the stress eigenvalues vanishes, that is when the rank of the stress tensor reduces to two and then one. This suggests that minimisation of bone density for a given stress intensity favours principal stresses with the same sign and as many zero principal stresses as possible. For instance, a uniaxial stress state would require a single aligned trabecula to minimise mass.

Unlike the empirical relationships postulated by Marangalou et al. (2015), quantitative relationships are derived between the stress and the fabric eigenvalues from the minimum density-to-stress intensity ratio $\lambda _{\rho }$ along three different criteria. It should be noted that in the presented analytical derivation, the correspondence of the fabric and stress eigenvalues may not necessarily follow the ordering relation and depends on the repartition of signs among the stress eigenvalues.45Comparison of the solutions by different criteria 20, 21 leads to quantitatively distinct but qualitatively resembling solutions. In particular, the 3D solutions for CFE and PSE overlap in the negative hydrostatic quadrant. The microstructural adaptation mechanism developed by Huiskes et al. (2000) combined with the concept of poroelastic fluid flow in the lacuno-canalicular compartment (Cowin 1999) as a primary mechanism for bone mechano-transduction favours the choice of a symmetric criterion. Indeed, the symmetric strain energy density is often used as stimulus at the ECM level and inspired the normalised CFE used in this work at the homogenised level. Nevertheless, the principal strain criterion can also be made symmetric and provides similar results as the CFE. As stated in the introduction, the criteria must not necessarily be identical for the resorption/formation activity around the lazy zone and the resorption associated with damage of the lacuno-canalicular network for excessive strains. It could be speculated here that two distinct criteria may have to be used for these processes: a first symmetric criterion that reflects fluid flow in the principal fabric directions and a non-symmetric criterion that describes the occurrence of ECM damage and the resulting resorption due to interruption of mechano-sensation.

### Inverse problem

No previous study investigating the inverse problem as an optimal fabric at the local level could be found. From a mathematical point of view, the inverse problem is hampered by the fact that the fabric tensor is positive definite and the stress tensor is not. In terms of eigenvalues, the domain of the latter is therefore 8 times "larger" and an isomorphism could only be sought on the absolute value of these eigenvalues. For a given fabric and set-point, multiple stress tensors from different octants are admissible and reach a local minimum, but only one such stress tensor from the hydrostatic octant reaches the global minimum for the entire fabric domain. As reflected in the forward problem, different stress tensors may lead to the same unique optimal fabric tensor and inversion of these relationships is obviously not univocal.

A qualitative agreement is found for the solutions of the stress ratios among the three considered criteria and the ones of CFE and PSE are even equivalent in both 2D and 3D. As imposed, the density to stress intensity ratios intersect at $m_1=m_2=m_3$ and the GYC solution is the lowest in both 2D and 3D.

Interestingly, beyond the alignment of principal fabric, stresses and strains, the hydrostatic solution with three negative stress and strain eigenvalues remains optimal, i.e. exhibits the highest stress intensity for a given bone density, for all the mechanostats and all fabric configurations. This finding coincides with the recognised fact that the musculoskeletal system favours compression of bone tissue in all loading directions. Tensile strains are tolerated up to the limit of the lower damage threshold, but is less optimal with regard to the non-symmetric optimisation criteria (GYC & PSE). However, for CFE as a symmetric metric, the opposed solution with three positive stress and strain eigenvalues is equivalent.

### General

The derived optimal solutions for all three mechanostats support Wolff’s original observation according to which the trabecular network follows the principal stresses in the proximal femur. This finding corresponds also to the minimal compliance of an orthotropic material in structural optimisation (Diaz and Lipton 1997). The present work provides a static optimisation framework to interpret not only the alignment of bone fabric with principal apparent stresses but also the relationship between the degree of anisotropy of fabric with respect to the anisotropy of the apparent stresses and the assignment of stress to fabric eigenvalues. This optimisation framework can be perceived as the best spatial arrangement of the available bone tissue for a given external stress tensor and, in the case of the CFE mechanostat, matches the intention of microstructural adaptation models that drive towards a homogeneous distribution of strain energy density at the tissue level (Huiskes et al. 2000). The appropriate density of bone is then determined by the set-point of the selected mechanostat like in the classical isotropic bone adaptation models (Carter et al. 1987).

Unlike the original fabric–elasticity and fabric–yield relationships models by Cowin (1985, 1986), the role of density and fabric in the fabric–elasticity and fabric–yield relationships models used in this work is assumed to be uncoupled. This is consistent with the normalisation of the fabric tensor, and the lack of statistically significant correlation between density and the degree of anisotropy. This uncoupling is an essential property for the design of the optimality problems minimising the bone density over stress intensity ratio for the different mechanostats.

An alternative mechanostat proposed previously is the trace of the strain tensor (Turner et al. 1997), the infinitesimal change of volume, that is described by two parallel surfaces along the trisectrix in the principal strain space and can vanish for non-trivial strain tensors. While attractive in terms of fluid flow, convexity, and equivalent to the principal strain criteria in 1D, it is not bound in terms of principal strain amplitudes in 2D or 3D. For this reason, it was not pursued in this work.

Since the density function is monotonic and extends to 1, the above results may also be applied to transverse isotropic or orthotropic cortical bone, but will require a refinement of the density functions $f(\rho )$ and $\hat{f}(\rho )$ as well as a distinct calibration of the fabric tensor $\textbf{M}$, which is work in progress. Moreover, the specialisation of the solutions to a fabric tensor of rank 2 can be applied to a thin cortical shell that does not experience strains in the normal direction and does not necessarily undergo adaptation along this axis. The 1D solution with a single principal strain applies to the loading of a single trabecula loaded along its axis under homeostatic conditions and relates the present work to the original mechanostat theory formulated by Frost (2003).

### Limitations

Several limitations of this work are now discussed.

The results of this study were derived for simple fabric–elasticity or fabric–yield relationships and illustrated only for specific values of the material parameters. Nevertheless, the findings can be applied to the generalised relationships in Zysset (2003); Schwiedrzik et al. (2013) by transforming the original fabric tensor $\textbf{M}$ into the following

46 $$\begin{aligned} \hat{\textbf{M}}=\frac{3}{\textrm{tr} \textbf{M}^l}\textbf{M}^l \end{aligned}$$

The eigenvalue ratios $\hat{m_i} / \hat{m_j}$ computed with the above method could then be transformed back into $m_i / m_j=(\hat{m_i} / \hat{m_j})^{1/ l}$. This also applies if the fabric tensor is measured with other methods than MIL such as MSL or GST.

This work relies on a continuum description associated with the existence of a bone RVE which morphology and mechanical descriptors represent a spatial average of the underlying microstructure and micro-mechanical variables. Bone morphology is approximated by density and fabric, while apparent strains and stresses are the homogenised versions of the micro-structural fields. The selected fabric–elasticity and fabric–yield relationships are phenomenological but proved successful in trabecular bone for the description of anisotropic elastic and yield properties (Maquer et al. 2015; Musy et al. 2017). Despite the meaningful correspondence between CFE and microscopic strain energy density, apparent properties of trabecular bone depend on boundary conditions (Pahr and Zysset 2008) and the micro-structural interpretation of the proposed macroscopic strain criteria should be clarified in the spirit of the work by Bachmann et al. (2023).

The optimal solutions presented in this work apply at the local level of an RVE. A forward bone adaptation algorithm for a whole bone structure can be derived from these solutions following the FE methods available in the literature (Carter and Beaupre 2001). The rates of density and of fabric can be driven by the difference between their current and optimum values at all integration points using two first-order differential equations that account for some coupling due to the same underlying remodelling process. Conversely, an inverse bone adaptation algorithm to retrieve an optimal set of load cases for an existing bone structure at homeostasis can be designed by minimising the sum of a specific distance between the applied stresses and the optimal stresses derived in this work over all integration points of the FE model for a given distribution of density and fabric. However, a proper time averaging procedure to compute a functional stress tensor rather than a scalar stimulus from multiple non-proportional load cases remains to be developed for practical implementation of the presented findings which will be the object of future work. Nevertheless, independently of the latter requirement, the benefit of exploiting fabric in the global inverse adaptation problem at the continuum level may be substantial since together with density, the eigenvectors and eigenvalues of the fabric tensor contribute to determine an optimal stress tensor to be compared with the stress produced by the candidate load cases. Eventually, the selection of the most realistic mechanostat/strain metric and appropriate rates for density and fabric will have to be established on experimental grounds.

### Conclusion

The solutions of forward and inverse optimisation problems of bone adaptation were investigated at the RVE level for three mechanostat criteria using fabric–elasticity and fabric–yield relationships. The optimality conditions were derived analytically, while the solutions were obtained numerically and illustrated by stress and fabric eigenvalue ratios for orientation/anisotropy and by density-to-stress intensity ratios for amplitude.

With the addition of meaningful time-averaging procedures for the functional stress tensor, the obtained results of the forward problem will allow efficient computation of anisotropic bone adaptation, while the results of the inverse problem will enable the estimation of bone loading using HR-pQCT or PCCT images that provide both density and fabric *in vivo*.

As stated in Zadpoor (2013), the most important question regarding bone tissue adaptation models is "do they faithfully and accurately represent the actual process of bone tissue adaptation". Regrettably, this work hardly contributes to the answer, but the proposed mechanostats including the distinct solutions for tension and compression will hopefully contribute to the interpretation of future *in vivo* experiments.

## Supplementary Information

Below is the link to the electronic supplementary material.

Supplementary file 1 (pdf 1623 KB)
