# Supplementary material for: Forward and inverse optimality problems of bone adaptation at the homogenised RVE level
Source: Biomech Model Mechanobiol. 2026 Jan 13;25(1):18. doi: 10.1007/s10237-025-02024-8 (PMC12799671; doi:10.1007/s10237-025-02024-8)
Supplement: Supplementary file 1 — (pdf 1623 KB) [file 10237_2025_2024_MOESM1_ESM.pdf]

Forward and Inverse Optimality Problems of Bone Adaptation at the Homogenised RVE Level

Philippe K. Zysset

ARTORG Center for Biomedical Engineering Research,
University of Bern, Switzerland

Supplementary Material

This supplementary material contains the resolution and the existence/uniqueness discussion of the optimality problems for the forward and inverse problems and for each criterion with the exception of the inverse problem for PSE which resolution is trivial. For clarity, the numbering of the sections follows the one of the main paper. The equation and figure numbers have the prefix letter "S".

---

3. Forward Problem

3.1. Complementary free energy density

3.1.1. Resolution

The minimum of the scalar function in Eq. 19 can be obtained by constructing the following Lagrange function with the constraint $\mathrm{tr}\mathbf{M} = 3$

$$L_\psi(\hat{\mathbf{S}}; \mathbf{M}, \lambda_\psi) = \widehat{\psi^*}(\hat{\mathbf{S}}; 1, \mathbf{M}) - \lambda_\psi(\mathrm{tr}\mathbf{M} - 3) \tag{S.1}$$

where λ_ψ is the Lagrange multiplier. A stationary point is looked for that satisfies

$$\begin{aligned} \nabla_{\mathbf{M}} L_\psi(\hat{\mathbf{S}}; \mathbf{M}, \lambda_\psi) = \nabla_{\mathbf{M}} \widehat{\psi^*}(\hat{\mathbf{S}}, 1, \mathbf{M}) - \lambda_\psi \mathbf{I} &= \mathbf{0} \\ \frac{\partial L_\psi}{\partial \lambda_\psi} = -(\mathrm{tr}\mathbf{M} - 3) &= 0 \end{aligned} \tag{S.2}$$

The gradient of the complementary free energy can be calculated analytically for the original fabric-elasticity relationship

$$\nabla_{\mathbf{M}} \widehat{\psi^*}(\hat{\mathbf{S}}; 1, \mathbf{M}) = \nabla_{\mathbf{M}} \mathbf{M}^{-1} \, \nabla_{\mathbf{M}^{-1}} (\frac{1}{2} \hat{\mathbf{S}} : \mathbb{E}(1, \mathbf{M}) \hat{\mathbf{S}}) \tag{S.3}$$

where $\nabla_{\mathbf{M}} \mathbf{M}^{-1} = -\mathbf{M}^{-1} \underline{\overline{\otimes}} \mathbf{M}^{-1}$ and

$$\mathbb{E}(1, \mathbf{M}) = -\frac{\nu}{\epsilon} (\mathbf{M}^{-1} \otimes \mathbf{M}^{-1}) + \frac{(1 + \nu)}{\epsilon} (\mathbf{M}^{-1} \underline{\overline{\otimes}} \mathbf{M}^{-1}) \tag{S.4}$$

[1]Email address of corresponding author: philippe.zysset@unibe.ch
ORCID: 0000-0002-4712-7047

In fact,

$$\nabla_{\mathbf{M}^{-1}}\frac{1}{2}(\hat{\mathbf{S}}:(\mathbf{M}^{-1}\otimes\mathbf{M}^{-1})\hat{\mathbf{S}} = \mathrm{tr}(\mathbf{M}^{-1}\hat{\mathbf{S}})\hat{\mathbf{S}} \tag{S.5}$$

$$\nabla_{\mathbf{M}^{-1}}\frac{1}{2}(\hat{\mathbf{S}}:(\mathbf{M}^{-1}\underline{\overline{\otimes}}\mathbf{M}^{-1})\hat{\mathbf{S}}) = \hat{\mathbf{S}}\mathbf{M}^{-1}\hat{\mathbf{S}} \tag{S.6}$$

Then

$$\nabla_{\mathbf{M}}\frac{1}{2}(\hat{\mathbf{S}}:(\mathbf{M}^{-1}\otimes\mathbf{M}^{-1})\hat{\mathbf{S}} = -\mathrm{tr}(\mathbf{M}^{-1}\hat{\mathbf{S}})\mathbf{M}^{-1}\mathbf{S}\mathbf{M}^{-1} \tag{S.7}$$

$$\nabla_{\mathbf{M}}\frac{1}{2}(\hat{\mathbf{S}}:(\mathbf{M}^{-1}\underline{\overline{\otimes}}\mathbf{M}^{-1})\hat{\mathbf{S}}) = -\mathbf{M}^{-1}\hat{\mathbf{S}}\mathbf{M}^{-1}\hat{\mathbf{S}}\mathbf{M}^{-1} \tag{S.8}$$

Using

$$(\mathbf{M}^{-1}\underline{\overline{\otimes}}\mathbf{M}^{-1})(\mathbf{M}\underline{\overline{\otimes}}\mathbf{M}) = \mathbf{I}\underline{\overline{\otimes}}\mathbf{I} \tag{S.9}$$

We obtain

$$(\mathbf{M}^{-1}\underline{\overline{\otimes}}\mathbf{M}^{-1})^{-1}\mathbf{I} = \mathbf{M}^2 \tag{S.10}$$

The Lagrangian equations S.2 reduce to

$$\begin{aligned} \frac{\nu}{\epsilon}\mathrm{tr}(\mathbf{M}^{-1}\hat{\mathbf{S}})\hat{\mathbf{S}} - \frac{(1+\nu)}{\epsilon}\hat{\mathbf{S}}\mathbf{M}^{-1}\hat{\mathbf{S}} - \lambda_\psi \mathbf{M}^2 &= \mathbf{0} \\ \mathrm{tr}\mathbf{M} - 3 &= 0 \end{aligned} \tag{S.11}$$

The tensorial equation has indeed a solution if and only if $\hat{\mathbf{S}}$ and $\mathbf{M}$ commute and therefore share all their eigenspaces. In other words, if the eigenvectors of fabric are aligned with the ones of the stress tensor [1].

Exploiting the common spectral decomposition of $\hat{\mathbf{S}}$ and $\mathbf{M}$, we obtain 4 scalar equations for the 3 fabric eigenvalues and the multiplier λ_ψ

$$\begin{aligned} \frac{\nu}{\epsilon}\left(\sum_k \frac{\hat{\sigma}_k}{m_k}\right)\hat{\sigma}_i - \frac{(1+\nu)}{\epsilon}\frac{\hat{\sigma}_i^2}{m_i} - \lambda_\psi m_i^2 &= 0 \qquad i=1,2,3 \\ \sum_k m_k &= 3 \end{aligned} \tag{S.12}$$

Using the convention $|\hat{\sigma}_1| \leq |\hat{\sigma}_2| \leq |\hat{\sigma}_3|$ so that the stress ratios σ_1/σ_3 and σ_2/σ_3 are contained in $[-1,1]$ the problem reduces to two unknown fabric ratios m_1/m_3 and m_2/m_3. The solution of Eq. S.12 was solved with Mathematica (v13.2, Wolfram Research Inc. Champaign, Illinois).

3.1.2. Existence and unicity

The free energy density function with respect to $\mathbf{M}$ can be calculated in the direct strain formulation

$$\begin{aligned} \widehat{\psi^*}(\hat{\mathbf{S}};1,\mathbf{M}) &= \hat{\mathbf{S}}:\left(-\frac{\nu}{\varepsilon}\mathbf{M}^{-1}\otimes\mathbf{M}^{-1} + \frac{1+\nu}{\varepsilon}\mathbf{M}^{-1}\underline{\overline{\otimes}}\hat{\mathbf{M}}^{-1}\right)\hat{\mathbf{S}} \\ &= \hat{\mathbf{S}}\mathbf{M}^{-1}:\left(-\frac{\nu}{\varepsilon}\mathbf{I}\otimes\mathbf{I} + \frac{1+\nu}{\varepsilon}\mathbf{I}\underline{\overline{\otimes}}\mathbf{I}\right)\mathbf{M}^{-1}\hat{\mathbf{S}} \end{aligned} \tag{S.13}$$

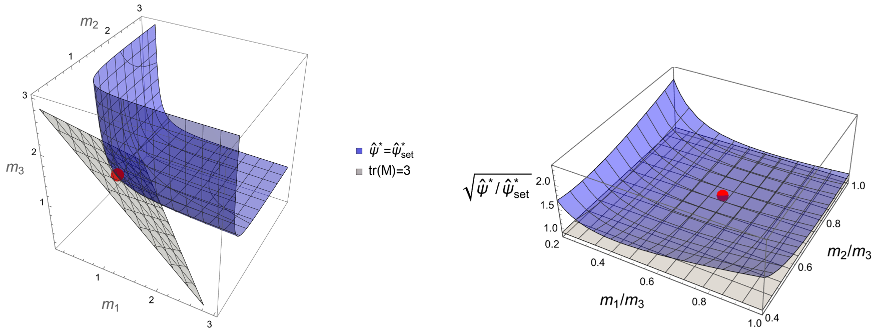

Figure S1: In the fabric space (left), the solution represents the tangent of the normalised complementary free energy density (CFE) with the normalisation of the fabric eigenvalues tr$\mathbf{M} = 3$. Unicity of the solution derives from convexity of the CFE with respect to fabric (right).

This represents a quadratic form for $\hat{\mathbf{S}}\mathbf{M}^{-1}$ which convexity relies on the same conditions as the isotropic stiffness tensor $\lambda \mathbf{I} \otimes \mathbf{I} + 2\mu \mathbf{I} \underline{\overline{\otimes}} \mathbf{I}$, that is $\lambda > 0$ and $\mu > 0$ when all strain eigenvalues differ from zero. This suggests that the complementary free energy is not only convex in $\mathbf{S}$, but also in $\mathbf{M}$ when all stress eigenvalues are non-zero. Accordingly, existence and unicity of the solution of the Lagrangian problem can be claimed.

3.2. Generalised yield criterion

3.2.1. Resolution

The minimum of this smooth function of $\mathbf{M}$ can be obtained by constructing a Lagrangian function with the constraint

$$L_y(\hat{\mathbf{S}}, \mathbf{M}) = y(\hat{\mathbf{S}}; 1, \mathbf{M}) - \lambda_y(\mathrm{tr}\mathbf{M} - 3) \tag{S.14}$$

and looking for its stationary point with respect to $\mathbf{M}$ and λ

$$\nabla_{\mathbf{M}} L_y(\hat{\mathbf{S}}, \mathbf{M}) = \nabla_{\mathbf{M}} y(\hat{\mathbf{S}}; 1, \mathbf{M}) - \lambda_y \mathbf{I} \quad = \quad \mathbf{0} \tag{S.15}$$

$$\frac{\partial L_y}{\partial \lambda_y} = -(\mathrm{tr}\mathbf{M} - 3) \quad = \quad 0 \tag{S.16}$$

The gradient of the yield function can be calculated as follows

$$\nabla_{\mathbf{M}} y(\hat{\mathbf{S}}; 1, \mathbf{M}) = \frac{1}{2\sqrt{\hat{\mathbf{S}} : \mathbb{F}(1, \mathbf{M})\hat{\mathbf{S}}}} \nabla_{\mathbf{M}}(\hat{\mathbf{S}} : \mathbb{F}(1, \mathbf{M})\hat{\mathbf{S}}) + \nabla_{\mathbf{M}}(\mathbf{F}(1, \mathbf{M}) : \hat{\mathbf{S}}) \tag{S.17}$$

Recalling

$$\mathbf{F}(1, \mathbf{M}) \quad = \quad f_0 \mathbf{M}^{-2} \tag{S.18}$$

$$\mathbb{F}(1, \mathbf{M}) \quad = \quad F_0^2(-\zeta_0\, \mathbf{M}^{-2} \otimes \mathbf{M}^{-2} + (\zeta_0 + 1)\mathbf{M}^{-2} \underline{\overline{\otimes}} \mathbf{M}^{-2}) \tag{S.19}$$

with

$$f_0 = \frac{1}{2}(\frac{1}{\sigma_0^+} - \frac{1}{\sigma_0^-}) \qquad F_0 = \frac{1}{2}(\frac{1}{\sigma_0^+} + \frac{1}{\sigma_0^-}) \tag{S.20}$$

Using

$$\nabla_{\mathbf{M}}\mathbf{M}^{-2} = -\mathbf{M}^{-1}\overline{\underline{\otimes}}\mathbf{M}^{-2} - \mathbf{M}^{-2}\overline{\underline{\otimes}}\mathbf{M}^{-1} \tag{S.21}$$

and

$$\nabla_{\mathbf{M}^{-2}}(\hat{\mathbf{S}} : (\mathbf{M}^{-2} \otimes \mathbf{M}^{-2})\hat{\mathbf{S}}) = 2\mathrm{tr}(\mathbf{M}^{-2}\hat{\mathbf{S}})\hat{\mathbf{S}} \tag{S.22}$$

$$\nabla_{\mathbf{M}^{-2}}(\hat{\mathbf{S}} : (\mathbf{M}^{-2}\overline{\underline{\otimes}}\mathbf{M}^{-2})\hat{\mathbf{S}}) = 2\hat{\mathbf{S}}\mathbf{M}^{-2}\hat{\mathbf{S}} \tag{S.23}$$

we obtain

$$\nabla_{\mathbf{M}}(\mathbf{M}^{-2} : \hat{\mathbf{S}}) = -(\mathbf{M}^{-1}\hat{\mathbf{S}}\mathbf{M}^{-2} + \mathbf{M}^{-2}\hat{\mathbf{S}}\mathbf{M}^{-1}) \tag{S.24}$$

$$\nabla_{\mathbf{M}}(\hat{\mathbf{S}} : (\mathbf{M}^{-2} \otimes \mathbf{M}^{-2})\hat{\mathbf{S}}) = -2\mathrm{tr}(\mathbf{M}^{-2}\hat{\mathbf{S}})(\mathbf{M}^{-1}\hat{\mathbf{S}}\mathbf{M}^{-2} + \mathbf{M}^{-2}\hat{\mathbf{S}}\mathbf{M}^{-1}) \tag{S.25}$$

$$\nabla_{\mathbf{M}}(\hat{\mathbf{S}} : (\mathbf{M}^{-2}\overline{\underline{\otimes}}\mathbf{M}^{-2})\hat{\mathbf{S}}) = -2\mathbf{M}^{-2}\hat{\mathbf{S}}\mathbf{M}^{-2}\hat{\mathbf{S}}\mathbf{M}^{-1} - 2\mathbf{M}^{-1}\hat{\mathbf{S}}\mathbf{M}^{-2}\hat{\mathbf{S}}\mathbf{M}^{-2} \tag{S.26}$$

By composing left and right by $\mathbf{M}$, the first Lagrangian equation becomes

$$\begin{aligned} & \frac{F_0^2}{\sqrt{\hat{\mathbf{S}} : \mathbb{F}\hat{\mathbf{S}}}}(\zeta_0 \mathrm{tr}(\mathbf{M}^{-2}\hat{\mathbf{S}})(\hat{\mathbf{S}}\mathbf{M}^{-1} + \mathbf{M}^{-1}\hat{\mathbf{S}}) \\ - & (1+\zeta_0)(\mathbf{M}^{-1}\hat{\mathbf{S}}\mathbf{M}^{-2}\hat{\mathbf{S}} + \hat{\mathbf{S}}\mathbf{M}^{-2}\hat{\mathbf{S}}\mathbf{M}^{-1})) \\ - & f_0(\hat{\mathbf{S}}\mathbf{M}^{-1} + \mathbf{M}^{-1}\hat{\mathbf{S}}) - \lambda_y \mathbf{M}^2 = \mathbf{0} \end{aligned} \tag{S.27}$$

This equation has a solution if and only if $\hat{\mathbf{S}}$ and $\mathbf{M}$ commute and therefore share their spectral decomposition. In other words, if the axes of fabric are aligned with the ones of the stress tensor.

We obtain a scalar equation for the fabric eigenvalues

$$\begin{aligned} \frac{2F_0^2}{\sqrt{\hat{\mathbf{S}} : \mathbb{F}(1,\mathbf{M})\hat{\mathbf{S}}}} \left(\zeta_0 (\sum_k \frac{\hat{\sigma}_k}{m_k^2}) \frac{\hat{\sigma}_i}{m_i} - (1+\zeta_0)\frac{\hat{\sigma}_i^2}{m_i^3} \right) - 2f_0 \frac{\hat{\sigma}_i}{m_i} - \lambda_y m_i^2 &= 0 \\ i &= 1, ...3 \\ \sum_k m_k &= 3 \end{aligned} \tag{S.28}$$

The solutions for the fabric ratios were computed with Mathematica (v13.2, Wolfram Research Inc, Champaign, Illinois).

3.2.2. Existence and unicity

We start from the equivalence of the damage function y with a convex quadratic form ([2]):

$$y(\mathbf{S}; 1, \mathbf{M}) + 1 = (\mathbf{S} - \mathbf{A}(\mathbf{M})) : \mathbb{A}(\mathbf{M})(\mathbf{S} - \mathbf{A}(\mathbf{M})) \tag{S.29}$$

where $y_{set} = 1$ and $\rho = 1$ without loss of generality.
Recalling

$$\mathbb{A} = \frac{\mathbb{F} - \mathbf{F} \otimes \mathbf{F}}{1 + \mathbf{A} : (\mathbb{F} - \mathbf{F} \otimes \mathbf{F})\mathbf{A}} \qquad \mathbf{A} = -\frac{1}{2}(\mathbb{F} - \mathbf{F} \otimes \mathbf{F})^{-1}\mathbf{F} \tag{S.30}$$

and realising that

$$\begin{aligned} y(\mathbf{S};\mathbf{M}) + 1 &\propto (\mathbf{S} - \mathbf{A}) : (\mathbb{F} - \mathbf{F} \otimes \mathbf{F})(\mathbf{S} - \mathbf{A}) \\ &= -(F_0^2\zeta_0 + f_0^2)\ (\mathbf{S} - \mathbf{A}) : \mathbf{M}^{-2} \otimes \mathbf{M}^{-2}(\mathbf{S} - \mathbf{A}) \\ &+ F_0^2(\zeta_0 + 1)\ (\mathbf{S} - \mathbf{A}) : \mathbf{M}^{-2} \underline{\overline{\otimes}} \mathbf{M}^{-2}(\mathbf{S} - \mathbf{A}) \\ &= -(F_0^2\zeta_0 + f_0^2)\ (\mathbf{S} - \mathbf{A})\mathbf{M}^{-2} : (\mathbf{I} \otimes \mathbf{I})\mathbf{M}^{-2}(\mathbf{S} - \mathbf{A}) \\ &+ F_0^2(\zeta_0 + 1)\ (\mathbf{S} - \mathbf{A})\mathbf{M}^{-2} : (\mathbf{I} \underline{\overline{\otimes}} \mathbf{I})\mathbf{M}^{-2}(\mathbf{S} - \mathbf{A}) \end{aligned} \tag{S.31}$$

Convexity of the yield function in terms of fabric $\mathbf{M}$ is again determined by the eigenvalues of the fourth-order tensor :

$$-(F_0^2\zeta_0 + f_0^2)(\mathbf{I} \otimes \mathbf{I}) + F_0^2(\zeta_0 + 1)(\mathbf{I} \underline{\overline{\otimes}} \mathbf{I}) \tag{S.32}$$

The existence and unicity of the Lagrangian problem can therefore be claimed for $-1 < \zeta_0 < \zeta_{0,crit}$ (Fig. S2).

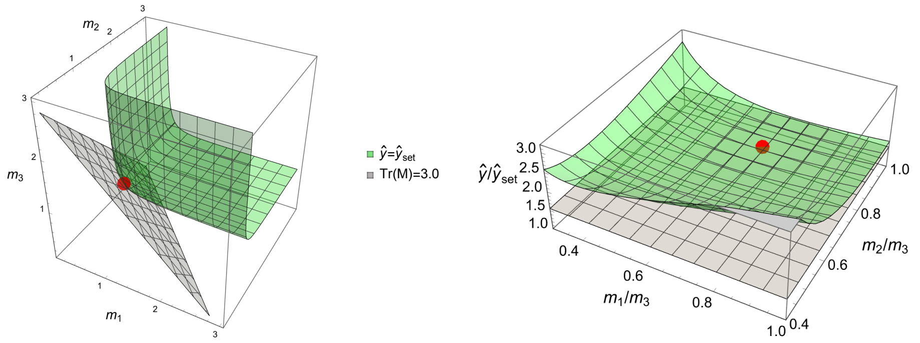

Figure S2: Left: in the fabric space, the solution represents the tangent of the normalised, generalised yield criterion (GYC) with the normalisation of the fabric eigenvalues tr$\mathbf{M}$ = 3. Right: particular solution for a given stress ratio. Unicity of the solution derives from convexity of the GYC with respect to fabric.

3.3. Principal strains

3.3.1. Resolution

The normalisation of the stress tensor $\hat{\mathbf{S}}$ is combined with the density function $f(\rho)$ into a parameter λ_ρ and the sought fabric tensor $\overline{\mathbf{M}}$ is defined by the equation

$$\mathbb{E}(1, \overline{\mathbf{M}})\hat{\mathbf{S}} = \lambda_\rho{}^{set}\mathbf{E} \qquad \hat{\mathbf{S}} = \frac{1}{\lambda_S}\mathbf{S} \qquad \lambda_\rho = \frac{f(\rho)}{\lambda_S} > 0 \tag{S.33}$$

Using

$$\mathbb{E}(1, \mathbf{M})\mathbf{S} = -\frac{\nu}{\epsilon}(\mathbf{M}^{-1} : \mathbf{S})\mathbf{M}^{-1} + \frac{(1+\nu)}{\epsilon}(\mathbf{M}^{-1}\mathbf{S}\mathbf{M}^{-1}), \tag{S.34}$$

we obtain the equation

$$-\frac{\nu}{\epsilon}(\mathbf{M}^{-1} : \hat{\mathbf{S}})\mathbf{M}^{-1} + \frac{(1+\nu)}{\epsilon}(\mathbf{M}^{-1}\hat{\mathbf{S}}\mathbf{M}^{-1}) = \lambda_\rho {}^{set}\mathbf{E} \qquad \text{(S.35)}$$

We obtain again 4 scalar equations for the 3 fabric eigenvalues and the factor λ_ρ

$$\begin{aligned} -\frac{\nu}{\epsilon}(\sum_k \frac{\hat{\sigma}_k}{m_k})\frac{1}{m_i} + \frac{(1+\nu)}{\epsilon}\frac{\hat{\sigma}_i}{m_i^2} - \lambda_\rho {}^{set}E_i &= 0 \\ i &= 1,2,3 \\ \sum_k m_k &= 3 \end{aligned} \qquad \text{(S.36)}$$

The resulting fabric depends on the principal stress quadrant and is not invariant with respect to a change of sign $\overline{\mathbf{M}}(\mathbf{S}) \neq \overline{\mathbf{M}}(-\mathbf{S})$ unless ${}^{set}E_- = {}^{set}E_+$.
The sought density is eventually determined by

$$\overline{\rho} = f^{-1}(\lambda_\rho \lambda_S) \qquad \text{(S.37)}$$

Using this set-point, the optimal fabric is the one that maintains the strain tensor at one of the eight corner of a hexahedron in principal strain space (Fig. S3). The quadrant of the stress eigenvalues determines which corner is achievable with a positive definite fabric tensor.

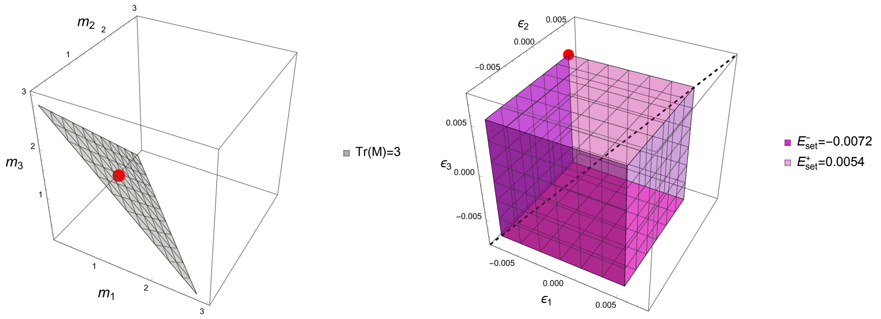

Figure S3: The set-point in the strain space is achieved at a corner of the cuboid for a given stress $\lambda_\rho = 0.0345$ and $\{\hat{\sigma}_1, \hat{\sigma}_2, \hat{\sigma}_3\} = \{-\frac{6}{17}, \frac{27}{17}, \frac{18}{17}\}$. The fabric solution is $\{m_1, m_2, m_3\} = \{0.83, 1.22, 0.95\}$. Note that the order relation of the fabric eigenvalues is not necessarily the same as the one of the stress eigenvalues.

3.3.2. Existence and uniqueness

Existence of a solution emerge from the quadratic form of the scalar equations obtained when multiplying all the terms by m_i^2.

$$-\lambda_\rho {}^{set}E_i m_i^2 - \frac{\nu}{\epsilon}(\sum_k \frac{\hat{\sigma}_k}{m_k})m_i + \frac{(1+\nu)}{\epsilon}\hat{\sigma}_i = 0 \qquad \text{(S.38)}$$

The discriminant of the quadratic form for m_i is

$$\Delta = (\frac{\nu}{\epsilon}(\sum_k \frac{\hat{\sigma}_k}{m_k}))^2 + 4\lambda_\rho \frac{(1+\nu)}{\epsilon} {}^{set}E_i \hat{\sigma}_i \tag{S.39}$$

Since the terms λ_ρ and $\frac{(1+\nu)}{\epsilon}$ are positive, the discriminant is positive in all cases when the signs of the corresponding stress eigenvalue and prescribed strain level coincide ($\hat{\sigma}_i$ and ${}^{set}E_i$). The equation has two roots and given that $-\lambda_\rho {}^{set}E_i \frac{(1+\nu)}{\epsilon}\hat{\sigma}_i$ is negative, there is a negative and a single admissible positive root for each fabric eigenvalue. This means that the solution corresponds to the prescribed strain levels which signs are identical to the one of the given stress eigenvalue.

4. Inverse Problem

4.1. Normalised complementary free energy density (CFE)

4.1.1. Resolution

A Lagrangian function is defined that is composed of the function to minimise and the constraint

$$L_\psi(\hat{\mathbf{S}}; \mathbf{M}, \lambda_\psi) = \widehat{\psi^*}(\hat{\mathbf{S}}; 1, \mathbf{M}) - \lambda_\psi(\mathrm{tr}|\hat{\mathbf{S}}| - 3) \tag{S.40}$$

where λ_ψ is the Lagrange multiplier and an absolute value of the stress tensor and its trace are defined by

$$|\hat{\mathbf{S}}| = \sum_{k=1}^{3} |\hat{\sigma}_k|(\boldsymbol{\sigma}_k \otimes \boldsymbol{\sigma}_k) \qquad \mathrm{tr}|\hat{\mathbf{S}}| = \sum_{k=1}^{3} |\hat{\sigma}_k| \tag{S.41}$$

A stationary point is looked for that satisfies

$$\begin{aligned} \nabla_{\hat{\mathbf{S}}} L_\psi(\hat{\mathbf{S}}; \mathbf{M}, \lambda_\psi) = \nabla_{\hat{\mathbf{S}}} \widehat{\psi^*}(\hat{\mathbf{S}}, 1, \mathbf{M}) - \lambda_\psi \mathbf{J}(\hat{\mathbf{S}}) &= \mathbf{0} \\ \frac{\partial L_\psi}{\partial \lambda_\psi} = -(\mathrm{tr}|\hat{\mathbf{S}}| - 3) &= 0 \end{aligned} \tag{S.42}$$

where

$$\mathbf{J}(\hat{\mathbf{S}}) = \sum_{k=1}^{3} \frac{\hat{\sigma}_k}{|\hat{\sigma}_k|}(\boldsymbol{\sigma}_k \otimes \boldsymbol{\sigma}_k) \tag{S.43}$$

is the gradient of $\mathrm{tr}|\hat{\mathbf{S}}|$ with respect to $\hat{\mathbf{S}}$.

In fact, the gradient of the CFE at $\rho = 1$ is the elastic strain produced by a given normalised stress tensor

$$\nabla_{\hat{\mathbf{S}}} \widehat{\psi^*}(\hat{\mathbf{S}}; 1, \mathbf{M}) = \mathbb{E}(1, \mathbf{M})\hat{\mathbf{S}} \tag{S.44}$$

Back to the stationary equations, we obtain

$$\begin{aligned} \mathbb{E}(1, \mathbf{M})\hat{\mathbf{S}} - \lambda_\psi \mathbf{J}(\hat{\mathbf{S}}) &= \mathbf{0} \\ \mathrm{tr}|\hat{\mathbf{S}}| - 3 &= 0 \end{aligned} \tag{S.45}$$

Accordingly,

$$\hat{\mathbf{S}} = \lambda_\psi \mathbb{E}^{-1}(1, \mathbf{M})\mathbf{J}(\hat{\mathbf{S}}) = \lambda_\psi \mathbb{S}(1, \mathbf{M})\mathbf{J}(\hat{\mathbf{S}}) \tag{S.46}$$

under the constraint $\mathrm{tr}|\hat{\mathbf{S}}| - 3 = 0$.
Using the compact form of the fabric-elasticity relationships,

$$\mathbb{S}(1, \mathbf{M}) = \lambda_0(\mathbf{M} \otimes \mathbf{M}) + 2\mu_0(\mathbf{M}\underline{\overline{\otimes}}\mathbf{M}), \tag{S.47}$$

we obtain

$$\hat{\mathbf{S}} = \lambda_\psi(\lambda_0 \mathrm{tr}(\mathbf{MJ})\mathbf{M} + 2\mu_0 \mathbf{MJM}). \tag{S.48}$$

Since $\hat{\mathbf{S}}$ and $\mathbf{J}(\hat{\mathbf{S}})$ have the same spectral decomposition, they are both aligned with the fabric tensor $\mathbf{M}$.
In this common coordinate system, the scalar equations become

$$\begin{aligned} \hat{\sigma}_i &= \lambda_\psi(\lambda_0(\sum_{l=1}^{3} m_l \frac{\hat{\sigma}_l}{|\hat{\sigma}_l|})m_i + 2\mu_0 \frac{\hat{\sigma}_i}{|\hat{\sigma}_i|} m_i^2) \qquad i = 1, 2, 3 \\ \sum_l |\hat{\sigma}_l| &= 3 \end{aligned} \tag{S.49}$$

The solution can be explored octant by octant where the sign of the stress eigenvalue is known. For instance, in the positive octant $+++$,

$$\begin{aligned} \hat{\sigma}_i^{+++} &= \lambda_\psi((\lambda_0 + 2\mu 0)m_i^2 + \lambda_0(m_i m_j + m_i m_k)) \qquad i = 1, 2, 3 \\ \sum_l |\hat{\sigma}_l| &= 3 \end{aligned} \tag{S.50}$$

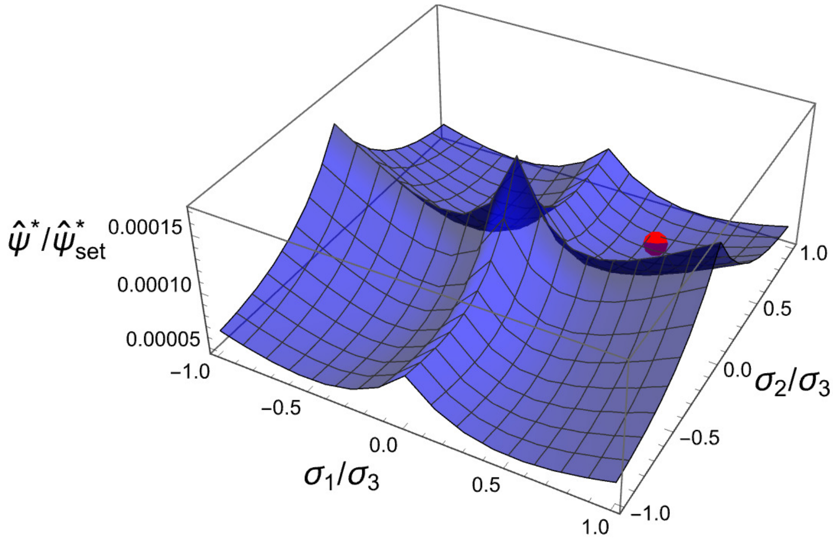

Figure S4: The relative CFE function for values of the fabric ratios that are representative of human trabecular bone. The overall minimum is located in the positive ratio octant, and this was observed for the entire domain of fabric ratios.

4.1.2. Existence

In the above fully positive octant example, and also in the symmetric fully negative octant, a solution exists for any combination of m_k and λ_ψ scales the intensity to satisfy the normalisation (Fig. S4). However, in other octants, involving negative and positive principal stresses, a stationary minimum may not necessarily be reached and the actual minimum would be located on the boundary. Accordingly, the stationary minima were computed for all octants and the global minimum identified numerically (Mathematica, Wolfram Inc, USA).

4.2. Generalised yield criterion (GYC)

4.2.1. Resolution

The Lagrangian of the stated problem is composed of the GYC function to minimise and the normalisation constraint

$$L_y(\hat{\mathbf{S}}; \mathbf{M}, \lambda_y) = y(\hat{\mathbf{S}}; 1, \mathbf{M}) - \lambda_y(\mathrm{tr}|\hat{\mathbf{S}}| - 3) \tag{S.51}$$

where λ_y is the Lagrange multiplier and we use the same definition for the absolute value of the stress tensor S.41. A stationary point is looked for that satisfies

$$\begin{aligned} \nabla_{\hat{\mathbf{S}}} L_y(\hat{\mathbf{S}}; \mathbf{M}, \lambda_y) = \nabla_{\hat{\mathbf{S}}} y(\hat{\mathbf{S}}, 1, \mathbf{M}) - \lambda_y \mathbf{J}(\hat{\mathbf{S}}) &= \mathbf{0} \\ \frac{\partial L_y}{\partial \lambda_y} = -(\mathrm{tr}|\hat{\mathbf{S}}| - 3) &= 0 \end{aligned} \tag{S.52}$$

where $\mathbf{J}(\hat{\mathbf{S}}) = \nabla_{\hat{\mathbf{S}}} \mathrm{tr}|\hat{\mathbf{S}}|$.

The gradient of the yield criterion is

$$\nabla_{\hat{\mathbf{S}}} y(\hat{\mathbf{S}}; 1, \mathbf{M}) = \frac{\mathbb{F}(1, \mathbf{M})\hat{\mathbf{S}}}{\sqrt{\hat{\mathbf{S}} : \mathbb{F}(1, \mathbf{M})\hat{\mathbf{S}}}} + \mathbf{F}(1, \mathbf{M}) \tag{S.53}$$

The stationary equations become

$$\begin{aligned} \frac{\mathbb{F}(1, \mathbf{M})\hat{\mathbf{S}}}{\sqrt{\hat{\mathbf{S}} : \mathbb{F}(1, \mathbf{M})\hat{\mathbf{S}}}} + \mathbf{F}(1, \mathbf{M}) - \lambda_y \mathbf{J}(\hat{\mathbf{S}}) &= \mathbf{0} \\ \mathrm{tr}|\hat{\mathbf{S}}| - 3 &= 0 \end{aligned} \tag{S.54}$$

Recalling

$$\mathbf{F}(1, \mathbf{M}) = f_0 \mathbf{M}^{-2} \tag{S.55}$$

$$\mathbb{F}(1, \mathbf{M}) = F_0^2(-\zeta_0\, \mathbf{M}^{-2} \otimes \mathbf{M}^{-2} + (1 + \zeta_0)\mathbf{M}^{-2} \underline{\overline{\otimes}} \mathbf{M}^{-2}) \tag{S.56}$$

Equations S.54 become

$$\hat{\mathbf{S}} = \sqrt{\hat{\mathbf{S}} : \mathbb{F}(1, \mathbf{M})\hat{\mathbf{S}}}\, \mathbb{F}^{-1}(\lambda_y \mathbf{J}(\hat{\mathbf{S}}) - \mathbf{F}) \tag{S.57}$$

under the implicit constraint $\mathrm{tr}|\hat{\mathbf{S}}| - 3 = 0$.

Using

$$\mathbb{F}^{-1} = \frac{1}{F_0^2}\left(\frac{\zeta_0}{(1+\zeta_0)(1-2\zeta_0)}\mathbf{M}^2 \otimes \mathbf{M}^2 + \frac{1}{(1+\zeta_0)}\mathbf{M}^2 \underline{\overline{\otimes}} \mathbf{M}^2\right) \tag{S.58}$$

We obtain

$$\begin{aligned}\hat{\mathbf{S}} = &\frac{\sqrt{\hat{\mathbf{S}} : \mathbb{F}(1,\mathbf{M})\hat{\mathbf{S}}}}{F_0^2}\left(\frac{\zeta_0}{(1+\zeta_0)(1-2\zeta_0)}\mathrm{tr}(\mathbf{M}^2(\lambda_y \mathbf{J}(\hat{\mathbf{S}}) - \mathbf{F}))\mathbf{M}^2\right.\\ &+ \left.\frac{1}{(1+\zeta_0)}\mathbf{M}^2(\lambda_y \mathbf{J}(\hat{\mathbf{S}}) - \mathbf{F})\mathbf{M}^2\right)\end{aligned} \tag{S.59}$$

Since $\hat{\mathbf{S}}$ and $\mathbf{J}(\hat{\mathbf{S}})$ have the same spectral decomposition, they must also be aligned with the fabric tensor $\mathbf{M}$ to expect a solution.
In this common coordinate system, the scalar equations become

$$\begin{aligned}\hat{\sigma}_i &= \frac{1}{F_0}\sqrt{\sum_{k=1}^{3}\frac{\hat{\sigma}_k^2}{m_k^4} - 2\zeta_0 \sum_{k<l=1}^{3}\frac{\hat{\sigma}_k\hat{\sigma}_l}{m_k^2 m_l^2}} \, * \\ &\quad \left(\frac{\zeta_0}{(1+\zeta_0)(1-2\zeta_0)}(\lambda_y \sum_{k=1}^{3} m_k^2 \frac{\hat{\sigma}_k}{|\hat{\sigma}_k|} - 3f_0)m_i^2\right. \\ &+ \left.\frac{1}{(1+\zeta_0)}(\lambda_y m_i^2 \frac{\hat{\sigma}_i}{|\hat{\sigma}_i|} - f_0)m_i^2\right) \\ i &= 1,2,3 \\ \sum_l |\hat{\sigma}_l| &= 3\end{aligned} \tag{S.60}$$

The solution can be explored again octant by octant where the sign of each stress eigenvalue is known. For instance, in the triple positive octant

$$\begin{aligned}\hat{\sigma}_i^{+++} &= \lambda_y((\lambda_0 + 2\mu 0)m_i^2 + \lambda_0(m_i m_j + m_i m_k)) \qquad i = 1,2,3 \\ \sum_l |\hat{\sigma}_l| &= 3\end{aligned} \tag{S.61}$$

4.2.2. Existence

In the above fully positive octant, an explicit solution exists for any fabric and the multiplier λ_y ensures that the linear constraint on the trace can be enforced. Nevertheless, this is not the case for different signs of the stress eigenvalues and stationary points were sought in all 8 octants. An example of a GYC function is shown in Fig. S5.

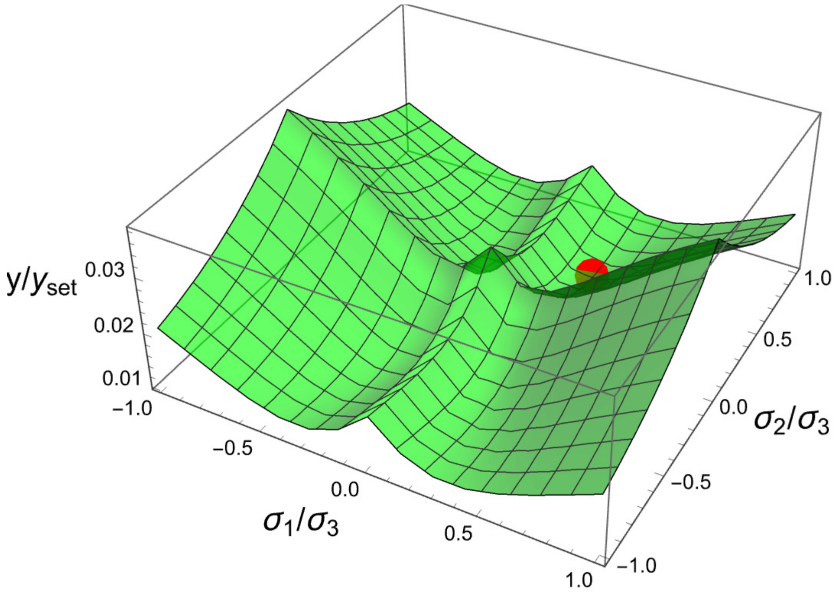

Figure S5: Example of a GYC function and its minimum for all negative signs of the stress eigenvalues using a representative value of m_2/m_3. Note that despite the graphical interpolation, the function is singular when $\sigma_1 = 0$ or $\sigma_2 = 0$.

References

[1] Z. P. Luo, K. N. An, A theoretical model to predict distribution of the fabric tensor and apparent density in cancellous bone, Journal of Mathematical Biology 36 (6) (1998) 557–568.

[2] J. J. Schwiedrzik, P. K. Zysset, An anisotropic elastic-viscoplastic damage model for bone tissue, Biomech Model Mechanobiol 12 (2) (2013) 201–13.
